# Targeting lysosomal HSP70 induces acid sphingomyelinase‐mediated disturbance of lipid metabolism and leads to cell death in T cell malignancies

**DOI:** 10.1002/ctm2.1229

**Published:** 2023-03-23

**Authors:** Yingjie Qing, Yongjian Guo, Qinwen Zhao, Po Hu, Hui Li, Xiaoxuan Yu, Mengyuan Zhu, Hongzheng Wang, Zhanyu Wang, Jingyan Xu, Qinglong Guo, Hui Hui

**Affiliations:** ^1^ State Key Laboratory of Natural Medicines Jiangsu Key Laboratory of Carcinogenesis and Intervention China Pharmaceutical University Nanjing China; ^2^ School of Pharmacy Nanjing University of Chinese Medicine Nanjing China; ^3^ School of Biopharmacy China Pharmaceutical University Nanjing China; ^4^ Department of Pharmacology School of Medicine and Holostic Integrative Medicine Nanjing University of Chinese Medicine Nanjing China; ^5^ Department of Hematology The Affiliated DrumTower Hospital of Nanjing University Medical School Nanjing China

**Keywords:** acid sphingomyelinase, heat shock protein 70, lipid metabolism, lysosomal membrane permeabilization, lysosome, T cell malignancies

## Abstract

**Background:**

T cell malignancies proliferate vigorously, are highly dependent on lysosomal function, with limited therapeutic options. Deregulation of lysosomal structure and function has been confirmed to be a key role in the treatment of hematologic malignant disease.

**Methods:**

Cell counting kit 8 and Annexin V/PI staining were used to assess the cell viability and apoptosis rate. Flow cytometry, liquid chromatography mass spectrometry, immunofluorescence and western blot were performed to detect the effect on lysosomes. Drug affinity responsive target stability, molecular docking and cellular thermal shift assay were employed to confirm the target protein of V8 on lysosomes. A xenograft model was constructed in *NOD/SCID* mice to assess the effect and mechanism.

**Results:**

V8, a new lysosomotropic compound, could be rapidly trapped by lysosomes and accumulation in lysosomes, contributing to lysosomal‐dependent cell death by evoking lysosomal membrane permeabilization (LMP), accompanied with disrupted lysosome and autophagic flux. Mechanistically, heat shock protein 70 (HSP70) was identified as the binding target of V8 in lysosome. As a downstream effect of targeting HSP70, enzymatic activity of acid sphingomyelinase (ASM) was inhibited, which induced disturbance of lipid metabolism, instability of lysosomal membrane, and leakage of cathepsin B and D, leading to LMP‐mediated cell death. In vivo study showed V8 well controlled the growth of the tumour and confirmed lysosomal cell death induced by V8.

**Conclusions:**

Collectively, this study suggests targeting lysosomal HSP70‐ASM axis by V8 illustrates the great value of drug therapy for T cell malignancies and the unlimited potential of lysosomal targeting for cancer therapy.

## Graphical abstract

- Find a new lysosomotropic drug V8.
- The characteristics of targeting lysosome HSP70 are clarified.
- Disturbance of lysosomal lipid metabolism is a new approach for T cell malignancies.
- The HSP70‐ASM axis regulates lysosomal stability.

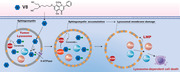

## 1 BACKGROUND

Lysosomes regulate cancer cell metabolism, differentiation, proliferation and death by maintaining a balance between anabolic and catabolic pathways.^1^ Due to nutrient limitations, tumour cells require higher activity and number of lysosomes to maintain physiological pathways such as autophagy, which makes lysosomes a sensitive weakness of tumours.^2^, ^3^, ^4^ As the volume and number of lysosomes in cancer cells increases, intracellular protons stored in lysosomes are significantly increased to maintain pH homeostasis.^3^ It was worth mentioning that the lysosomes of T cell malignancies differ greatly in content and activity from the lysosomes of normal cells making them potentially more amenable to lysosome‐targeting agents.^5^, ^6^ T‐cell malignancies are the general name of diseases with abnormal clonal proliferation of T lymphocytes at various stages, including acute T‐cell lymphoblastic leukemia (T‐ALL) and T‐cell non‐Hodgkin lymphoma.^7^ The main treatment methods of these diseases include radiotherapy, chemotherapy and stem cell transplantation, but it is usually accompanied by recurrence, drug resistance and toxic and side effects.^8^ Therefore, lysosomal targeted drugs which have great characteristic in accumulating in lysosomal acid lumen and induce lysosomal cell death (LCD) could be a therapeutic idea for T‐cell malignancies.^4^

Lipids play an important role in maintaining lysosome stability. Sphingomyelin (SM) is hydrolyzed into ceramide and phosphorylcholine by acid sphingomyelinase (ASM), a lysosomal glycoprotein.^9^ Lysosomal lipid metabolism and redox homeostasis are regulated in a bidirectional manner, so as to jointly maintain cell homeostasis.^10^, ^11^ The accumulation of compounds in the lysosomes' interior membranes lead to the disruption of lysosomal membrane proteins such as ASM etc.^12^, ^13^ ASM functional inhibition requires the drug to accumulate in the lysosomes and eventually lead to the accumulation of lipid like SM.^14^ SM and phospholipids are lipids that accumulate within late endosomal/lysosomal cell compartments due to disruption of lysosomal lipid homeostasis, which contributes to lysosomal membrane damage and lysosomal membrane permeabilization (LMP), resulting in the broken of cellular homeostasis and LCD.^13^

The upstream of ASM is regulated by heat shock protein 70 (HSP70).^15^, ^16^ The recent studies found HSP70 bound to bis(monoacylglycer)phosphate (BMP) and BMP‐ASM interaction was stabilized, resulting in lower lysosomal membrane SM levels and decreased membrane stability and induced LMP.^15^ Often, many functional ASM inhibitors are drugs that mainly interfere with other biological targets, such as tricyclic antidepressants. Therefore, affecting the activity of ASM by specifically targeting HSP70 on lysosomes can play a more accurate anti‐tumour effect.^14^ Multiple stimuli, including drugs, can induce LMP in tumour cells via HSP70 localization in their endosomal/lysosomal compartments.^17^ Targeting HSP70 in lysosomes could have an impact on the stability of the tumour‐specific subset of lysosomes, as well as lysosomal protease trafficking, which is the tumour's ‘achilles heel’.^18^ HSP70 inhibitors have been discovered in abundance, however selective inhibition remains a serious difficulty for cancer treatment. Thus, the selectivity for HSP70 of targeting lysosomes might be molecular‐spatial dual targeting advantages for the treatment of malignancies.

Lysosomal enzyme abnormalities and lysosomal storage disorders are commonly found in T cell malignancies, which provide a treatment method to destroy T‐cell malignancies while sparing normal hematopoietic cells.^1^, ^5^, ^6^, ^19^ Here, we identified a new compound V8, which was a lysosomotropic agent with the potential of drugability, induced lysosomal cell death via HSP70‐ASM axis. In vitro and vivo experiments proved V8 had the advantage of killing T cell malignancies. In order to verify the therapeutic value of targeting lysosomal HSP70 on T cell malignancies, we conducted the study on V8. In brief, our research introduced a new strategy for targeting lysosomes and selectively anti‐T cell malignancies.

## 2 MATERIALS AND METHODS

### 2.1 Compounds and reagents

V8 (C_24_H_29_NO_7_, MW: 443.49) with the purity ≧ 99% is provided by Prof. Zhiyu Li in China Pharmaceutical University. V8 was dissolved in dimethyl sulfoxide (DMSO) with a concentration of 0.02 M and stored at −80°C. Medium was used to dilute the working solution like RPMI‐1640 or DMEM, the final concentration of DMSO should not over 0.1%. In related studies, controls were treated with the same DMSO ratio. V8 injection was prepared by Prof. Xue Ke (China Pharmaceutical University) for intraperitoneal injection (the initial mother liquor concentration was 1.5 mg/mL, diluted with normal saline before using). Z‐VAD‐fmk (HY‐16658B), CQ (HY‐17589A), Rapamycin (HY‐10219), ML346 (HY‐18669), TRC051384 (HY‐101712), Bafilomycin A1 (HY‐100558), Ly93 (HY‐114307), and acridine orange (AO) hydrochloride (HY‐101879) were obtained from MedChem Express (MCE) (New Jersey, USA). Adriamycin (ADM) was obtained from Main Luck Pharm Inc, Shenzhen. β‐actin, GAPDH, HSP70, Cathepsin D (CTSD), Galectin‐3, caspase‐3, active caspase‐3, horse radish peroxidase (HRP) goat anti‐mouse/rabbit IgG (H+L) were obtained from ABclonal Technology (Wuhan, China). Cathepsin B (CTSB) and LC3 were obtained from CST Inc (MA, USA). ASM and PARP‐1 were obtained from Proteintech (CA, USA). p62 and LAMP2A were obtained from Abcam (Cambridge, UK). Lysosensor DND‐189 was purchased from YEASEN Biotech (Shanghai, China), 4′,6‐ diamidino‐2‐phenylin doledihydrochloride (DAPI) and 2,7‐dichlorodi‐hydrofluorescein diacetate (DCFH‐DA) were obtained from Beyotime Biotechnology (Nanjing, China). KeyGen Biotech (Nanjing, China) provided the Lysotracker RED. Goat anti‐Rabbit/Mouse IgG (H+L) Cross‐Adsorbed Secondary Antibody Fluor 488 (A‐11008 and A‐11001), Goat anti‐Rabbit/Mouse IgG (H+L) Cross‐Adsorbed Secondary Antibody Fluor 594 (R37117 and A‐11032), LipidTOX phospholipidosis detection regent (H34350), and BODIPY FL C12‐sphingomyelin (D7711) were purchased from ThermoFisher Scientific (MA, USA).

### 2.2 Cell culture

The human T‐ALL cell line (Jurkat cells), human T‐cell lymphoma cell lines (Hut‐102 and Hut‐78, Myla cells), human acute lymphoblastic leukemia cell line (Molt‐4 cells) and human normal colonic epithelial cell line (NCM460), human lymphatic endothelial cell line (HLEC), human renal tubular epithelial cell line (HK2), human embryonic kidney cell line (HEK293T) cells were purchased from National Collection of Authenticated Cell Cultures (Shanghai). Primary lymphoma cells from diagnosed patients (The Affiliated DrumTower Hospital of Nanjing University Medical School, Nanjing, China) and the lymphocyte‐monocyte separation medium (Jingmei, Nanjing, China) were used to collect peripheral blood mononuclear cells (PBMCs) from healthy donors. Jurkat, Hut‐102, and Hut‐78, Molt‐4, Myla, HLEC cells were cultured in RPMI‐1640 medium (Thermo Fisher Scientific, Gibco). NCM460, HK2, HEK293T cells were cultured in DMEM medium (Thermo Fisher Scientific, Gibco). 10% fetal bovine serum (FBS) (Thermo Fisher Scientific, Gibco) supplemented in the medium of cells and 100 U/mL of penicillin and 100 μg/mL streptomycin (Lukang Pharmaceutical, Jining, China) were added in the medium; cells were cultured in a humidified environment with 5% CO _2_ at 37°C.

### 2.3 Cell viability assay

Cell viability was determined by using CCK8 kit (KeyGen Biotech). 1.2 × 10^4^ cells per well were added in a 96‐well plate (100 μL medium), cells treated with V8 at various concentrations for 12 h. Incubation was carried out for 3 h at 37°C after the addition of 10 μL of CCK8, then measuring absorbance at 450 nm by using an Automatic Microplate Reader ELx800 (BioTek). 50% inhibitory concentration (IC_50_) was calculated following the previous study.^20^

### 2.4 Real time quantitative polymerase chain reaction (RT‐qPCR) analysis

RT‐qPCR was performed according to the previous study.^21^ The primer sequences were in Supplementary Materials and Methods.

### 2.5 Apoptosis detection

The cells were examined using a FITC‐labelled Annexin V/PI Detection Kit (KeyGen Biotech). Cell apoptosis was detected by using a FACS Calibur flow cytometer (BD Biosciences, CA, USA). Data were analyzed by using FlowJo software (Tree Star, Ashland, OR, USA).

### 2.6 AO staining

The cells were stained with AO before collecting and incubated at 37°C for 20 min. The fluorescence was detected under a laser confocal scanning microscope or flow cytometry (BD Biosciences). AO produces red fluorescence (E_m_: 650 nm) in lysosome, and green fluorescence (E_m_: 530 nm) in cytoplasm and nuclear.

### 2.7 Immunofluorescence

Immobilize cells in the 4% paraformaldehyde for 15 mins, then removed paraformaldehyde. Then permeabilized with Triton X‐100 for cells. After blocking with 3% bovine serum albumin (BSA) for 1 h, cells were incubated with primary antibody overnight at 4°C.The cells were treated for 1 h (h) at room temperature with the secondary antibody, DAPI dye for 5 mins. Using A confocal laser scanning microscope microscopy to capture images (FV1000; Olympus, Tokyo, Japan).

### 2.8 Transmission electron microscopy (TEM)

Jurkat cells were planted in dishes and given DMSO or V8 for the duration of the experiment. After collected and washed, 2.5% glutaraldehyde was added to a .1 μM phosphate buffer and the cells was fixed overnight at 4°C and incubated with osmium tetraoxide for 2 h at 4°C. Samples were dehydrated with upgraded ethanol (30%−95%), then epoxy resin was used to embed it (Solarbio Life Sciences, G8590). The ultrathin slices were then stained with lead citrate and 3% aqueous uranyl acetate. The cells were examined using a transmission electron microscope (Hitachi H‐7000FA, Tokyo, Japan).

### 2.9 Lysotracker RED and Lysosensor DND‐189 staining

Cells were labelled with LysoTracker Red (KeyGen Biotech) or Lysosensor DND‐189 (YEASEN Biotech) for 20 min at 37°C. Cells were then washed and fluorescence was detected under a flow cytometry (BD Biosciences).

### 2.10 LipidTOX Phospholipidosis assay

LipidTOX Green Phospholipidosis Detection Reagent (Thermo Fisher Scientific) was added to the cells for assessing phospholipid accumulation according to the previous study.^22^ Using confocal laser scanning microscope to capture images (FV1000; Olympus, Tokyo, Japan) with a ×60 magnification.

### 2.11 BODIPY FL C12‐Sphingomyelin staining

BODIPY FL C12‐Sphingomyelin (Thermo Fisher Scientific) and LysoTracker Red (KeyGen Biotech) was used to stain cells for tracking SM accumulation and localization according to the previous study.^22^ A confocal laser scanning microscope was used to capture the images (FV1000; Olympus, Tokyo, Japan) with a ×60 magnification.

### 2.12 ASM activity assay

After V8 treatment for indicated times, liquid nitrogen was used for multiple freezing and lysing cells. The lysate was centrifuged for 14000 rpm for 10 min, the supernatant was collected and measured protein concentrations. The activity of ASM was detected by using the Acid Sphingomyelinase Assay Kit (Fluorimetric) (Abcam) in accordance with the manufacturer's recommendations. Briefly, 20 μL sample was added to a 96‐well plate (black, flat bottom) and after 3 h of incubation at 37°C with 20 μL of sphingomyelin working solution, 20 μL of sphingomyelinase assay combination was added to each well. Signals detected by microplate reader (Thermo Fisher Scientific) correspond to the readings at Ex/Em = 540/590 nm (cut off at 570 nm) 2 h after incubation at room temperature.

### 2.13 Plasmid transfection and RNA interference

*CTSB*, *CTSD* shRNA and negative control (NC) constructs used pLV3ltr‐puroU6 vector system were obtained from Corues Biotechnology (Nanjing, China) for RNA interference. HEK293T cells were transfected with *CTSB* or *CTSD* shRNA constructs and NC along with a lentiviral cocktail and HG transgene reagents for 48 h following the manufacturer's instructions for the lentiviral packaging kit (YEASEN Biotech). Afterwards, the viral supernatant was collected. Then, the viral supernatant infects the target cells. Add 2 μg/mL puromycin to select shRNA to construct cells.

For constructing double knock down cell model by lentiviral vectors, *CTSB* shRNA construct created in PLV3ltr‐puroU6 vector system was purchased from Corues Biotechnology, *CTSD* shRNA construct created in pLV‐Hygro‐U6 vetor system, was purchased from VectorBuilder (Guangzhou, China). We first constructed *CTSB* shRNA cells, and then the virus supernatant of *CTSD* shRNA infected target *CTSB* shRNA cells, 250 μg/mL hygromycin B was added to determine whether *CTSB* shRNA cells expressed *CTSD* shRNA.

For transfection plasmid, mRFP‐GFP‐LC3 lentiviral vector plasmid was purchased from HanBio Technology (Shanghai, China). The vector was transfeted into Hut‐102 and Jurkat cells according to the previous study.^23^ GFP‐LC3 and mAG‐GAL3 plasmid were obtained from Addgene (Cambridge, MA, USA). Lipofectamine 3000 transfected cells with GFP‐LC3 plasmid (Invitrogen, Carlsbad, CA, United States). mAG‐GAL3 lentiviral vector plasmid was transfected into HEK293T cells for 48 h. Viral supernatant was then collected. The viral supernatant was used to infect Jurkat cells. 2 μg/mL puromycin was added to check for the expression of the mAG‐GAL3 construct cells.

### 2.14 Clustered regularly interspaced short palindromic repeats (CRISPR)/clustered regularly interspaced short palindromic repeats‐associated protein 9 (Cas9) genome editing

As mentioned, HSP70‐knockout cells were constructed by usingCRISPR/Cas9.^24^ In summary, the pLenti CRISPR/Cas9 V2 vector expressing sgRNA targeting HSP70 was transfected into 293T cells, viral fluid was to infect Jurkat cells, and HSP70‐knockout cells were screened with puromycin. Western blotting and genome DNA sequencing confirm successful knockout of HSP70 (Sanger sequencing in Supplemental methods and materials). The sequences of sgRNA are as followed, sgHSP70: CCTTTCCAGGTGATCAACGA, which was designed and synthesized by Corues Biotechnology.

### 2.15 Drug affinity responsive target stability (DARTS) assay

DARTS was carried out in the same way as the prior trial, with slight changes.^25^ Hut‐102 cells were taken out and then lysed by M‐PER (Thermo Fisher Scientific). After centrifugation, 1 × TNC buffer (50 mM Tris, 140 mM NaCl, 10 mM CaCl_2_) was added into cell lysates. M‐PER lysate was used to dilute samples and divided into two experimental and control groups. The experimental group was treated with 60 μM V8, and the control group was treated with equal volume DMSO, then rotated overnight at 4°C. The lysates were digested with pronase (Sigma‐Aldrich, Taufkirchen, German) of 1:100 for 30 min at 37°C, then protease inhibitor was added. Then adding loading buffer and protein was denatured in a boiling water bath. After electrophoresis, the gel was sent to process LCMS/MS detection, which was completed by Shanghai OE Biotech Co., Ltd (LM2021‐25234). In the proving experiment, western blot experiment was also used to detect the changes of target protein by adding different proportions of pronase (1:1000, 1:1500).

### 2.16 Cellular thermal shift assay (CETSA)

The methods are as described above.^26^ Briefly, Jurkat cells were incubated with V8 (2 μM) and DMSO for 1 h, respectively. Then, the mixture was divided into 100 μL aliquot in tubes and heated according to established procedure. Cells were lysed in Thermal Cycler, then liquid nitrogen was used for multiple freezing and lysing cells, then centrifuged for 20–‐25 min. After centrifugation, 4 × loading buffer was added then boiled and analyzed by Western blot.

### 2.17 Biolayer interferometry (BLI) analysis

The BLI analysis is based on the study reported.^27^ The procedure is repeated in a circular fashion for a total of four main steps, including loading, baseline, association and dissociation. ForteBio Octet data acquisition and analytics software captured and analyzed this data (Port Washington, USA). The experiment was conducted by Bio‐Lab Biotechnology Co. LTD (Wuhan, China).

### 2.18 Molecular docking

The crystal structure of HSP70 (PDB ID: 5TKY) was downloaded from protein data bank, and analyzed the interactions with V8 by Glide docking software. Details were in Supplementary Materials and Methods.

### 2.19 Western blot analysis

Cells treated with or without V8 for certain time were collected. Amersham Imager 600 was used for detection (GE Company, USA). Details were in Supplementary Materials and Methods.

### 2.20 Lysosomal isolation and LC‐MS methods

After cells were incubated with Perphenazine for indicated times, lysosome and cytosolic fractions of cells were performed using the Lysosome Isolation Kit (Invent Biotechnologies, Minnesota, USA) according to the following the methods reported by Kim and Ravodina et al.^28^, ^29^ The bicinchoninic acid method was used to quantify various cellular fractions. Supplementary Materials and Methods cover the LC‐MS technique conditions in detail.

### 2.21 Intracellular reactive oxygen species (ROS) assay

Cells were stained with DCFH‐DA. The ROS level was evaluated via the fluorescence intensity, which was detected by flow cytometry (BD Biosciences, CA, USA).

### 2.22 Xenograft tumour growth studies

Female *NOD/SCID* mice (5–6 weeks, 16∼20 g, Slaccas Shanghai Laboratory Animal Co., Shanghai, China) were exposed to radiation (1.5 Gy). Matrigel basement membrane matrix (Sigma‐Aldrich) was used to suspend Hut‐102 cells in serum‐free RPMI‐1640 media, which was added into cells to increase tumourigenicity. 5 × 10^6^ Hut‐102 cells were subcutaneously injected into each mouse. When the tumour volume reached 50−100 mm^3^, the mice were randomly divided into 5 groups (*n* = 4 per group): blank, control and V8 (5, and 10 mg/kg), ADM .5 mg/kg. 0.9% normal saline, V8, ADM was administered via intraperitoneal injection every 2 days for 2 weeks in control, V8 and ADM group respectively. Blank group is that control of observation under blank condition without any treatment. The tumour volume and body weight were monitored every 2 days. Tumour volume calculate method was according to previous study.^30^ At the end, the mice were euthanized and collected blood. Routine blood test was measured using a hematology analyzer (MEK‐7222K, Nihon Kohden). After 2 weeks, the mice were sacrificed, and the organs were stained for hematoxylin‐eosin (H&E) staining , the tumours were stained Ki‐67, LC3, Galectin‐3, LAMP1, CTSD.

### 2.23 Statistical analysis

The mean and standard error of the mean (SEM) are used to describe all of the results . The data comes from at least three separate parallel studies. Statistical analysis of multiple groups (data with normal distribution) was performed by one‐way analysis of variance followed by Turkey's test. Differences between two groups were determined using a Two‐tailed Student's *t*‐test. * *p* < 0.05 was represented significant, ** *p* < 0.01 and *** *p* < 0.001 were represented different levels of significant difference. GraphPad Prism 8.0 software was used to statistical analysis.

## 3 RESULTS

### 3.1 V8 induced cell death showed cell selectivity and was involved in lysosome

**FIGURE 1:**
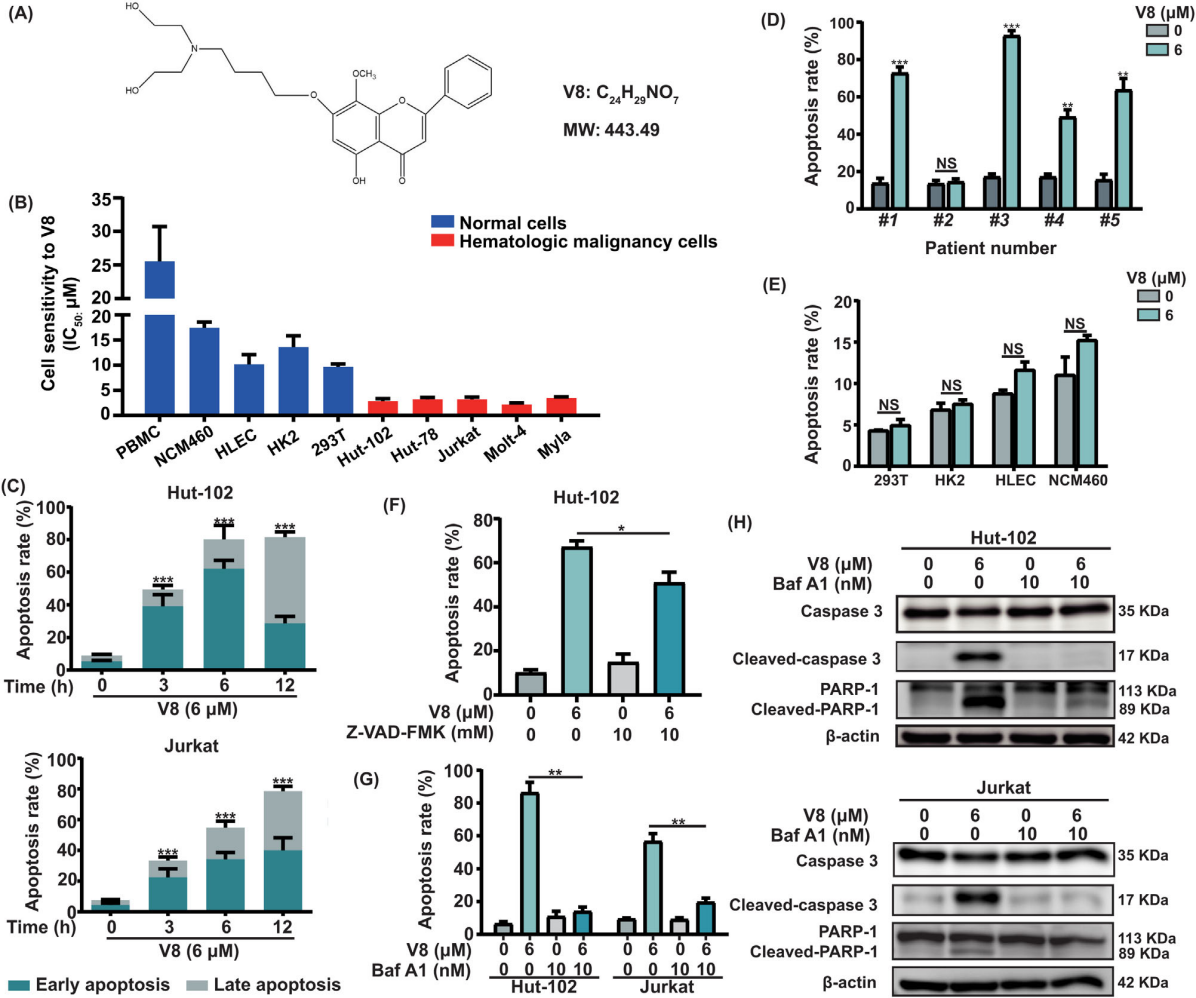
V8 induced cell death showed cell selectivity and was involved in lysosome. (A) Chemical structure of V8. (B) Various hematologic malignancy cells and normal cells were treated with indicated concentrations of V8 for 12 h. Cell sensitivity to V8 was assessed by IC_50_ (μM). Graphs show mean ± SEM values (*N* = 3). (C) Hut‐102 and Jurkat cells were treated with 6 μM V8 for 0, 3, 6, 12 h and Apoptosis rate (%) were determined (*N* = 3). (D) Apoptosis rates (%) were determined in primary T lymphoma cells treated with V8 for 6 μM for 12 h (*N* = 3). (E) Apoptosis rates (%) were determined in 293T, HK2, HLEC, NCM460 cells treated with V8 for 6 μM for 12 h (*N* = 3). (F) Apoptosis rate (%) were determined in Hut‐102 cells pretreated with or without Z‐VAD‐FMK (10 mM) for 1 h, and then treated with or without V8 (6 μM) for 6 h (*N* = 3). (G) Apoptosis rates (%) were determined in Hut‐102 and Jurkat cells pretreated with or without Baf A1(10 nM) for 1 h, and then treated with or without V8 (6 μM) for 6 h (*N* = 3). (H) Hut‐102 and Jurkat cells pretreated with or without Baf A1 (10 nM) for 1 h, and then treated with or without V8 (6 μM) for 6 h. The protein expression of PARP‐1, Caspase‐3 and its cleaved form were determined by western blot. β‐actin was used as loading controls. Three parallel experiments have yielded the mean (mean ± SEM, *N* = 3). * *p* < .05; ** *p* < .01; *** *p* < .001. NS, no statistically significant, compared with control group.

A diverse typological of hematologic malignancies and normal cell lines were used to assess the anticancer activity and the tumour‐selectivity ofV8. The chemical structure of V8 was shown in Figure 1A. Following a 12 h treatment with V8, cell cytotoxicity of V8 was identified in various hematologic malignancy cell lines, compared with human normal cell lines and PBMC, and the IC_50_ of V8 in hematologic malignancy cells were lower than in normal cells (Figure 1B, Figure S1A). V8 induced apoptosis with time and dose dependent in different T cell malignancies cell lines (Figure 1C, Figure S1B,C). In five cases of primary T cell malignancies samples, four of samples were induced significant apoptosis by the treatment of V8 (Figure 1D). However, V8 did not induce significant apoptosis in normal cell lines, which indicated the cell selectivity of V8 (Figure 1E). Consistent with apoptosis induction, cleaved PARP‐1 and Caspase 3 were dose‐dependent upregulated after treatment with V8 (Figure S1D). Pan‐Caspase inhibitor (Z‐VAD‐FMK) partly reversed V8‐induced apoptosis, indicating that Caspase‐dependent apoptosis was involved in V8 induced apoptosis (Figure 1F). When pretreatment with 10 nM Baf A1, a vacuolar‐ATPase (v‐ATPase) inhibitor, can induce the lysosomal alkalinization, reduced apoptosis and expression of cleaved PARP‐1 and Caspase 3 induced by V8 (Figure 1G,H, Figure S1E). To sum up, the results showed that V8 had the characteristics of tumour cytotoxicity and cell selectivity, and had the potential to against T‐cell malignancies.

### 3.2 V8 inhibited autophagic flux in T cell malignancies mediated by affecting the lysosomal function

**FIGURE 2:**
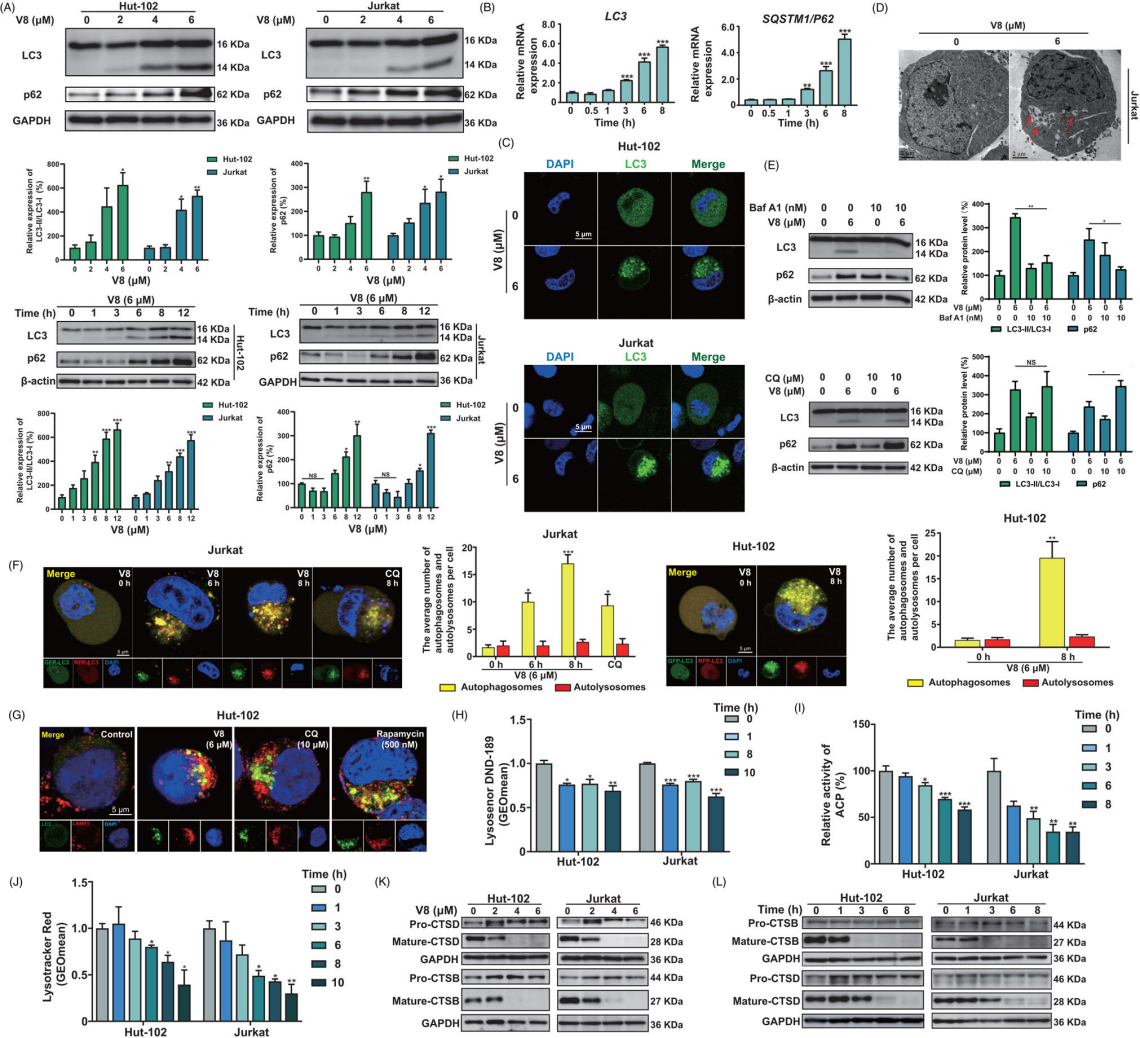
V8 inhibited autophagic flux in T cell malignancies mediated by affecting the lysosomal function. (A) Hut‐102 and Jurkat cells treated with 0, 2, 4, 6 μM V8 for 10 h or treated with 6 μM V8 for 0, 1, 3, 6, 8, 12 h. The protein expression of LC3, p62 was detected by western blot. GAPDH and β‐actin were used as loading controls (*N* = 3). (B) Hut‐102 cells were treated with 6 μM V8 for 0, 0.5, 1, 3, 6, 8 h. The relative mRNA level of *LC3*, *SQSTM1/P62* was measured by RT‐qPCR (*N* = 3). (C) The Hut‐102 and Jurkat cells were transfected with a GFP‐LC3 plasmid and treated with 6 μM V8 for 6 h. The LC3 puncta determined by laser confocal microscope (scale bar: 5 μm) (*N* = 3). (D) Jurkat cells were treated with V8 (6 μM) for 6 h. Representative microscopy images of Jurkat cells were obtained by TEM. The red arrow indicates autophagic vacuoles containing cytoplasmic context. Scale bar, 2 μm (*N* = 3). (E) Hut‐102 cells were treated with 6 μM V8 and Baf A1 (10 nM pretreated for 1 h) or CQ (10 μM pretreated for 1 h) for 6 h, and western blot was used to determine the expression of LC3 and p62 (*N* = 3). (F) Hut‐102 and Jurkat cells transfected with mRFP‐GFP‐LC3 plasmid. 6 μM V8 was treated for 0, 6, 8 h in Jurkat and for 8 h in Hut‐102 cells, 10 μM CQ was treated for 8 h in Jurkat cells. The LC3 puncta formation was detected by laser confocal microscope (scale bar: 5 μm) (*N* = 3). (G) Hut‐102 cells were treated with 6 μM V8, 10 μM CQ and 500 nM rapamycin for 6 h. The immunofluorescence analysis performed with LAMP1 (red), LC3 (green) and DAPI (blue). An analysis of overlay levels was conducted (scale bar: 5 μm) (*N* = 3). (H) Hut‐102 and Jurkat cells treated with 6 μM V8 for 0, 1, 8, 10 h, flow cytometry was used to detect lysosomal pH by staining cells with Lysosensor (*N* = 3). (I) Relative enzymatic activity of acid phosphatase (ACP) in Hut‐102 and Jurkat cells treated with V8 (6 μM) for 0, 1, 3, 6, 8 h (*N* = 3). (J) Hut‐102 and Jurkat cells treated with 6 μM V8 for 0, 1, 3, 6, 8, 10 h, Lysotracker Red was used to stain the cells and to detect fluorescence by using flow cytometry (*N* = 3). (K) Hut‐102 and Jurkat cells treated with 0, 2, 4, 6 μM V8 for 12 h. The protein expressions of CTSD and CTSB (including pro‐ and mature‐ form) were determined by western blot. GAPDH was used as loading control (*N* = 3). (L) Hut‐102 and Jurkat cells treated with 6 μM V8 for 0, 1, 3, 6, 8 h. The protein expressions of CTSD and CTSB (including pro‐ and mature‐ form) were determined by western blot. GAPDH was used as loading controls. Three parallel experiments have yielded the mean (mean ± SEM, *N* = 3). * *p* < .05; ** *p* < .01; *** *p* < .001, compared with control group.

Endocytic and autophagic processes use lysosomes as digesting organelles.^31^ Autophagy is a symbolic function of lysosomes, the state of autophagy can reflect lysosomes, so autophagy was detected.^32^ Thus, we confirmed the effect of V8 on autophagic condition. We observed the significant accumulation of autophagic vesicles in sustained treatment of V8, as the expression of LC3 and p62 increased translationally and transcriptionally (Figure 2A,B), number of GFP‐LC3 puncta increased in cytoplasm (Figure 2C, Figure S2A), and double‐membrane autophagosomes were identified in V8‐treated cells by TEM (Figure 2D). We further investigate autophagic condition by treating cells with late stage autophagic flux inhibitor CQ. The LC3‐II and p62 levels were significantly increased by CQ (10 μM) and V8 (6 μM) respectively in Hut‐102 cells. CQ treatment increased the accumulation of p62 caused by V8, but co‐incubation of cells with V8 and CQ did not result in an increase in LC3‐II levels when compared to V8 treatment alone (Figure 2E), indicating that V8 inhibited the degradation of autophagosomes. However, the increased expression of LC3‐II and p62 treatment with V8 were decreased when combined with Baf A1, it was speculated that Baf A1 affected the acidic environment of lysosomes (Figure 2E). To further evaluate autophagic flux,^33^ mRFP‐GFP‐LC3 lentiviral plasmid was transferred in Jurkat and Hut‐102 cells. As shown in figure 2F, the treatment with V8 increased the number of autophagosomes (yellow dots in merged images) in Jurkat and Hut‐102 cells. These data collectively indicated that V8 inhibited autophagic flux in T cell malignancies.

The inhibition of autophagic flux can be linked to either preventing autophagosome‐lysosome fusion or impaired autophagosome degradation.^34^ To figure out these two possibilities, we investigated the colocalization of LC3 and LAMP1 in Hut‐102 cells. Results showed that most LC3 puncta were colocalized with LAMP1 in V8 treated group, which was the same as Rapamycin treated group (Figure 2G). We also evaluated the colocalization of GFP‐LC3 puncta and LAMP1 in Jurkat cells. We found most GFP‐LC3 puncta were colocalized with LAMP1 in V8 treated group, which was similar to be observed in Hut‐102 cells (Figure S2B). It was indicated that V8 did not block autophagosome‐lysosome fusion in T cell malignancies. Therefore, we supposed V8 affected lysosomal degradation capacity by identifying lysosome acidification, lysosomal degradation capacity, and cathepsins mature through Lysosensor/Lysotracker staining,^35^, ^36^ activity of enzyme acid phosphatase (ACP),^34^ and the protein expressions of pro/mature‐cathepsins, respectively.^37^ Results showed that V8 decreased the fluorescence intensity of Lysosensor Green and Lysotracker Red, it was indicated that V8 reduced the acidity of the lysosomes (Figure 2H,J). V8 decreased the activity of ACP in a time‐dependent manner (Figure 2I). The cathepsin proteases are the most numerous hydrolases found in lysosomal compartments, and CTSB and CTSD are the major lysosomal proteases.^34^, ^38^ Mature‐CTSB and mature‐CTSD protein expression were reduced in a time and dose‐dependent manner (Figure 2K,L, Figure S2C,D). In summary, our data demonstrate that V8 inhibited lysosomal degradation activity, thereby leading to inhibition of autophagic flux.

### 3.3 V8 induced lysosomal membrane damage and permeabilization, promoting LCD

**FIGURE 3:**
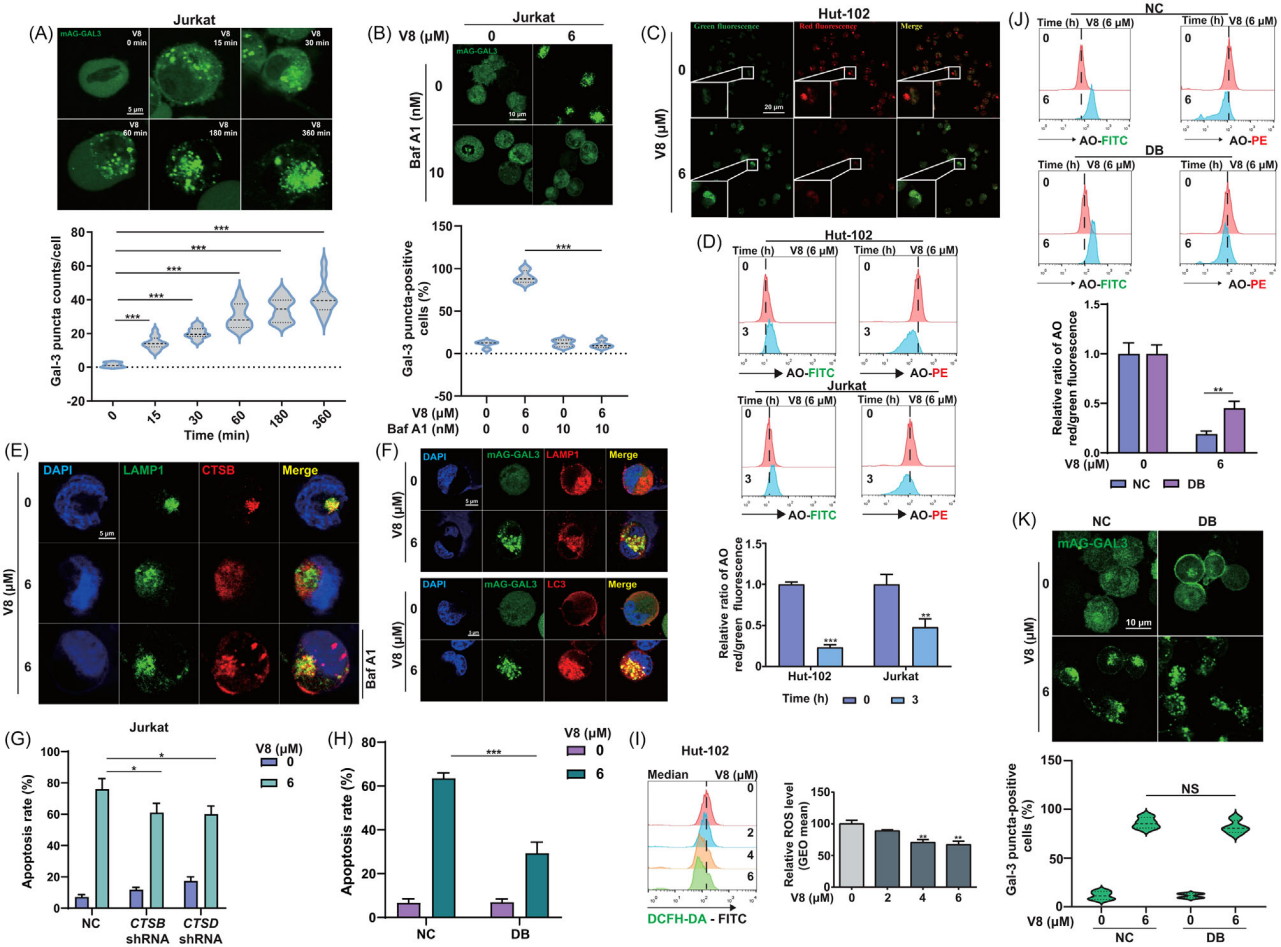
V8 induced lysosomal membrane damage and permeabilization, promoting lysosomal cell death (LCD). (A) Jurkat cells transfected with mAG‐GAL3 plasmid were treated with 6 μM V8 for indicated times and the Gal‐3 puncta were calculated (scale bar: 5 μm) (*N* = 12). (B) Jurkat cells transfected with mAG‐GAL3 plasmid were treated with 6 μM V8 and 10 nM Baf A1 (pretreated for 1 h) for 3 h and the ratio of Gal‐3 puncta‐positive cells were calculated (scale bar: 5 μm) (*N* = 4). (C) Laser confocal microscope was used to detect the acridine orange (AO) fluorescence in Hut‐102 cells after treating for 6 μM V8 for 3 h and staining with AO (*N* = 3). (D) Hut‐102 and Jurkat cells treated with 6 μM V8 for 3 h were stained with AO staining, AO fluorescence were detected by flow cytometric. Relative ration of AO Red/Green fluorescence was calculated (*N* = 3). (E) Hut‐102 cells were treated with 6 μM V8, 10 nM Baf A1 (pretreated for 1 h) for 6 h. The immunofluorescence analysis performed with LAMP1 (green), CTSB (red) and 4′,6‐ DAPI (blue). An analysis of overlay levels was conducted (scale bar: 5 μm) (*N* = 3). (F) Analysis of immunofluorescence used LAMP1 (red) or LC3 (red) and mAG‐GAL3 (green) in Jurkat cells. The cells were with V8 for 6 h (*N* = 3). (G) Apoptosis rate (%) was determined in *CTSB* shRNA and *CTSD* shRNA Jurkat cells compared with negative control (NC) via Annexin V/PI staining by flow cytometry (*N* = 3). (H) Apoptosis rate (%) was determined in DB cells compared with NC, the cells were stained with Annexin V/PI measured by flow cytometry (*N* = 3). (I) Hut‐102 cells were treated with V8 for 6 h, and ROS levels were determined by DCFH‐DA and flow cytometry (*N* = 3). (J) NC and DB cells treated with 6 μM V8 for 3 h. Before collection, the cells were stained with AO staining, and the AO fluorescence was measured using flow cytometry. Relative ration of AO Red/Green fluorescence was calculated (*N* = 3). (K) DB cells transfected with mAG‐GAL3 plasmid were treated with 6 μM V8 for 3 h, and Gal‐3 puncta‐positive cells were detected by immunofluorescence (scale bar: 10 μm). The ratio of Gal‐3 puncta‐positive cells was calculated. Three parallel experiments have yielded the mean (mean ± SEM, *N* = 3). * *p* < .05; ** *p* < .01; *** *p* < .001. NS, no statistically significant, compared with NC group.

Lysosomal membrane damage could be contributed to V8 disturbed lysosomal function. LGALS3/galectin‐3 is the best marker for the detection of lysosomal membrane damage.^39^ Gal‐3 puncta counts/cell increased in a time‐dependent manner after V8 treatment, indicating the damage of lysosomal membrane (Figure 3A). After pretreatment with Baf A1, Gal‐3 puncta‐positive cells were significantly decreased compared with single treatment with V8, which demonstrated the nature of lysosomotropic contributed to lysosomal membrane damage (Figure 3B). When lysosomal membrane damage is carried out to a certain extent, LMP can be induced.^40^, ^41^ Decreased AO‐labelled vesicles and AO red/green fluorescence after V8 treatment, indicating instability of lysosomal membrane and the occurrence of LMP (Figure 3C,D). CTSB was released from lysosomes induced by V8, which was inhibited by Baf A1, indicating V8‐induced LMP was dependent on lysosomal acidity (Figure 3E, Figure S3A). Colocalization of the Gal‐3 and LAMP‐1 and LC3 also indicated the damage of lysosomal membrane (Figure 3F, Figure S3B).

The release of cathepsin proteases into the cytoplasm as a result of LMP causes cell death via LCD.^40^ We supposed that the leakage of CTSB and CTSD was the reason of LCD. As knocking down *CTSB*/*CTSD* in Jurkat cells, V8‐induced apoptosis was inhibited (Figure 3G, Figure S4A). Double knocking down (DB) (*CTSB*, *CTSD* shRNA) apparently decreased V8‐induced apoptosis, indicating that CTSB and CTSD were co‐participated in LCD (Figure 3H, Figure S4B). Oxidative stress from high cellular ROS levels is one of the main factors leading to LMPs.^42^ To investigate whether V8‐triggered LMP is associated with ROS, we first analyzed intracellular ROS levels by DCFH‐DA, and the ROS levels were decreased with the treatment of V8, indicating that the LMP and LCD was not correlated with ROS levels (Figure 3I). However, *CTSB*, *CTSD* knockdown increased AO red/green fluorescence, indicating CTSB and CTSD promoted LMP (Figure 3J). However, *CTSB*, *CTSD* knockdown did not affect the ration of Gal‐3 puncta‐positive cells after treated with V8 (Figure 3K), which made the speculation that another component of lysosomal membranes regulates membrane fragility and promotes lysosomal membrane damage.

### 3.4 Identification of V8 as a potent lysosomotropic compound and HSP70 was a direct binding target on lysosome of V8

**FIGURE 4:**
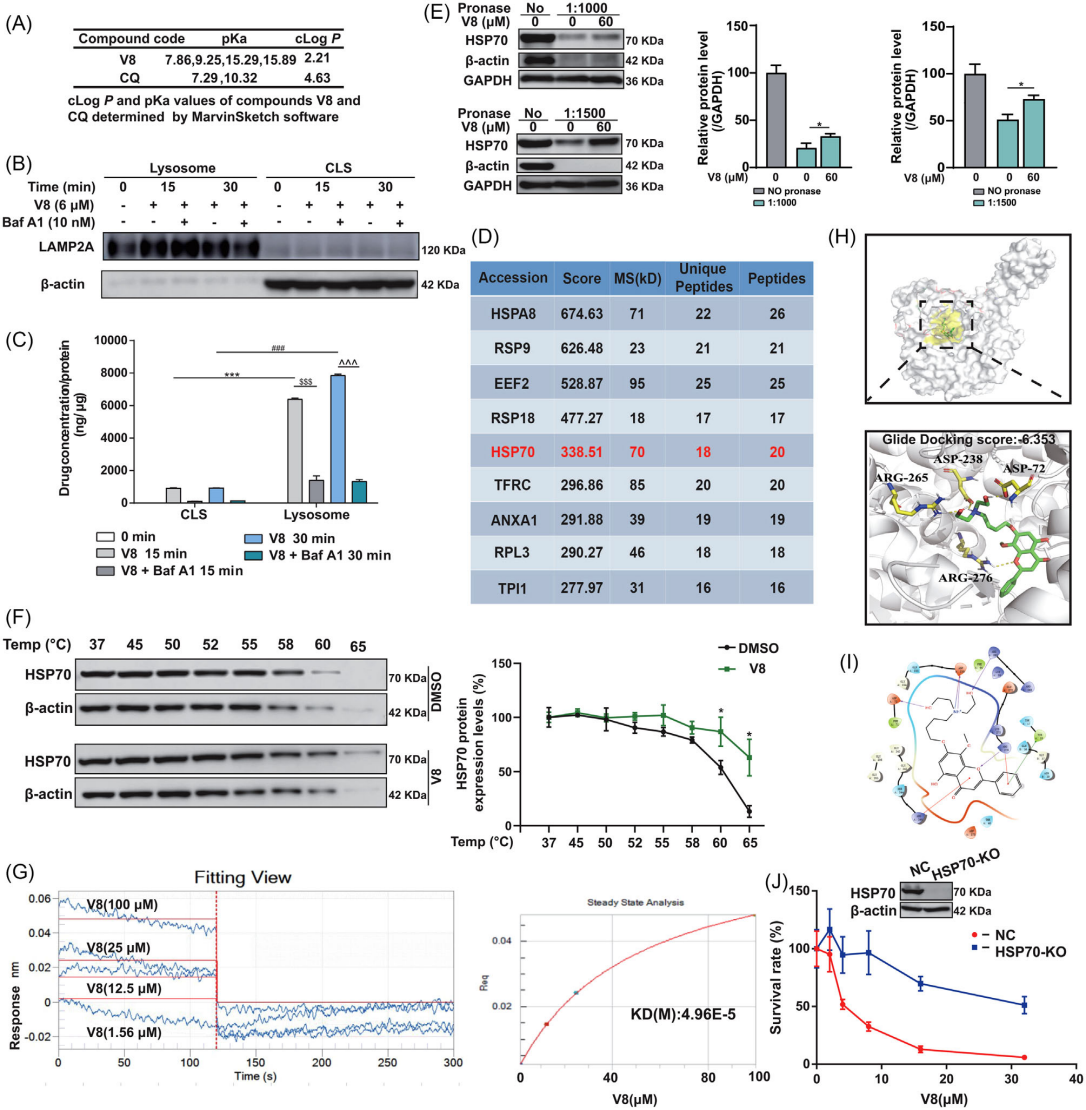
Identification of V8 as a potent lysosomotropic compound and HSP70 was a direct binding target on lysosome of V8. (A) Lipophilicity and basicity of the test compound V8 and CQ. (B) Jurkat cells were treated with phosphate buffered saline (PBS), 6 μM V8 for 15 and 30 min with or without pretreatment of 10 nM Baf A1 for 1 h. Cells were homogenized and fractionated to separate lysosomes and cytoplasm after lysosome separation (CLS). Immunoblotting against Lamp 2A, β‐actin protein confirmed separation of lysosomes (*N* = 3). (C) The relative concentrations of V8 in CLS and lysosomes (relative to the protein concentration) detected by liquid chromatography/mass spectrometry (LC/MS). *** *p* < 0.001 V8 lysosome versus CLS (15 min). ^###^ *p* < 0.001 V8 lysosome versus CLS (30 min). ^$$$^ *p* < 0.001 V8 lysosome versus V8 + Baf A1 lysosome (15 min). ^^^^^ *p* < 0.001 V8 lysosome versus V8 + Baf A1 lysosome (30 min) (*N* = 3). (D) LC–MS/MS analysis of top nine differential protein in drug affinity responsive target stability (DARTS) experiment. (E) Western Blot analysis of HSP70 in Hut‐102 treated by 60 μM V8 and pronase (1:1000 and 1:1500) (*N* = 3). (F) CETSA melt curve of HSP70 for heat treatment of Jurkat cells in the absence and in the presence of V8 (2 μM) (*N* = 3). (G) Real‐time kinetic binding sensorgrams of different concentrations of V8 increasing from 1.56 to 100 μM are shown. Response (nm) indicates the optical thickness on the biosensor layer. The equilibrium binding signal (Req) revealed by the flattened curve is reached (*N* = 3). (H and I) Molecule docking examination of V8 and HSP70 (PDB ID:5TKY) HSP70 binding mode (H) and 2D interaction map of V8 (I). (J) Western blot for HSP70 expression in control or stable HSP70 knockout (HSP70‐KO) Jurkat cells. CCK8 assay for the viability of control and HSP70‐KO Jurkat cells treated with V8 (0–32 μM). Three parallel experiments have yielded the mean (mean ± SEM, *N* = 3).

Accumulating literature suggest that lysosomotropic compounds are a type of weak base that can easily pass through the lipid bilayer, they enter acidic organelles and accumulate.^43^ Because protonated molecules have little retro‐diffusion (ion trapping), they become stuck and collect inside the acidic lumen.^44^ The degree of ion trapping depends on the compound's physicochemical properties such as pKa (logarithmic acid dissociation constant) and clog*P* value (a logarithmic partition coefficient).^45^ We evaluated the basicity and lipophilicity of the compound by using MarvinSketch software, and found that V8 is lipophilic, basic with more ionizable groups (Figure 4A). Moreover, in order to identify V8 possess the nature of lysosome trapping, we first assessed the accumulation of V8 in lysosomes compared with cytoplasm by the liquid chromatography and mass spectrophotometric (LC‐MS) method. Jurkat cells were treated with phosphate buffered saline, 6 μM V8 for 15 and 30 min with or without pretreatment of 10 nM Baf A1 for 1 h. Cells were sub‐fractionated to separate lysosomes and cytoplasm after lysosome separation (CLS). Western blot results showed LAMP2A, which is a lysosome marker, has demonstrated that lysosomes have been separated from whole cells (Figure 4B). LC‐MS experiments showed that V8 were accumulated in higher concentrations in lysosomes than in CLS after treatment for 15 and 30 min (Figure 4C). However, when pretreatment with Baf A1 for 1 h, the accumulation of V8 was dropped sharply compared with single‐treatment of V8 (Figure 4C). Acidic organelles are maintained by the v‐ATPase proton pump, which drives the accumulation of lysosomotropic drugs. Therefore, the uptake of lysosomotropic compounds is reduced by v‐ATPase inhibitors, such as Baf A1.^44^ According to these findings, V8 is a lysosomotropic compound.

Compounds with lysosomotropic properties alone do not necessarily produce lethal lysosomal membrane damage. The rapid and potent performance of V8 leads us to speculate that it has a specific target at the lysosomal site. Previous study has shown V8 was highly enriched in lysosomes and V8 disordered lysosomal membrane stability, suggesting the potential binding target was related to lysosome and we performed DARTS‐based quantitative proteomics approach by LC‐MS/MS analysis. To determine whether a protein is credible, the more peptides matched, the more unique pep, and the higher signal and score, the more credible it is. In order to improve the quality of data, this study set screening conditions for the identified candidate proteins: unique peptides greater than 15 and peptides greater than 15. Then, the order from high to low was carried out in the range of score sequence HT greater than 200, and finally nine proteins were screened, the report identified a list of putative direct binding proteins (Figure 4D). HSP70 reduces membrane fragility and act as the lysosome protective effect.^46^ Considering the existence facts of HSP70 and lysosome, HSP70 was screened out from the table and identified as a potent target of V8. Small‐molecule binding proteins were protected and enriched during proteolysis, we used western blot to reveal that increased stabilization of HSP70 during the proteolysis process when treated with V8 at 60 μM in Hut‐102 cells (Figure 4E). To confirm the binding of V8 and HSP70 in Jurkat cells, CETSA proved that V8 could increase the thermal stability of HSP70 in Jurkat cells at 60°C (Figure 4F). To further validate the binding affinity of HSP70 and V8, BLI was used to confirm the binding activity. The binding effect of V8 to HSP70 was investigated by employing increasing concentrations of V8 (1.56, 12.5, 25, 100 μM) to monitor the real‐time association/dissociation of V8 with HSP70. The result showed that V8 had a fast binding and fast dissociation with HSP70, which was revealed by the concentration‐dependent increase in response indicating the optical thickness (nm) on the sensor layer (Figure 4G). In addition, figure 4G showed that the kinetic constants including dissociation affinity (KD) calculated by the ForteBio data analysis software was 4.96E‐5 M.

To further investigate the possible binding pattern between V8 and HSP70 domain, we performed a molecular docking examination. Glide Binding score indicated an ideal docking score (−6.353), and the subsequent MMGBSA calculation exhibited the binding free energy (△G) of the compound with −49.19 kcal/mol (Figure 4H). As was shown in Figure 4I, V8 binds to HSP70 through both polar and hydrophobic interactions. Asp72, Asp238, Arg265 and Arg276 are the major contributors for polar interactions, while The40, Phe44, Gly37, Gly234 and Gly343 are mainly involved in hydrophobic interactions. All these results showed V8 direct binding to HSP70 on lysosome. To confirm that V8‐induced tumour inhibition is indeed via targeting HSP70, we established HSP70 stable knockout (HSP70‐KO) Jurkat cells by using CRISPR/Cas9 system. HSP70‐KO and negative control (NC) Jurkat cells were treated with different concentrations of V8, and the cell viability was measured by CCK8 assay. We found that HSP70‐KO Jurkat cells were very insensitive to the treatment of V8 (Figure 4J), suggesting that the anticancer activity of V8 is indeed through targeting HSP70.

### 3.5 V8 disrupted lipid metabolism and disordered lysosomal membrane stability mediated by HSP70‐ASM axis

**FIGURE 5:**
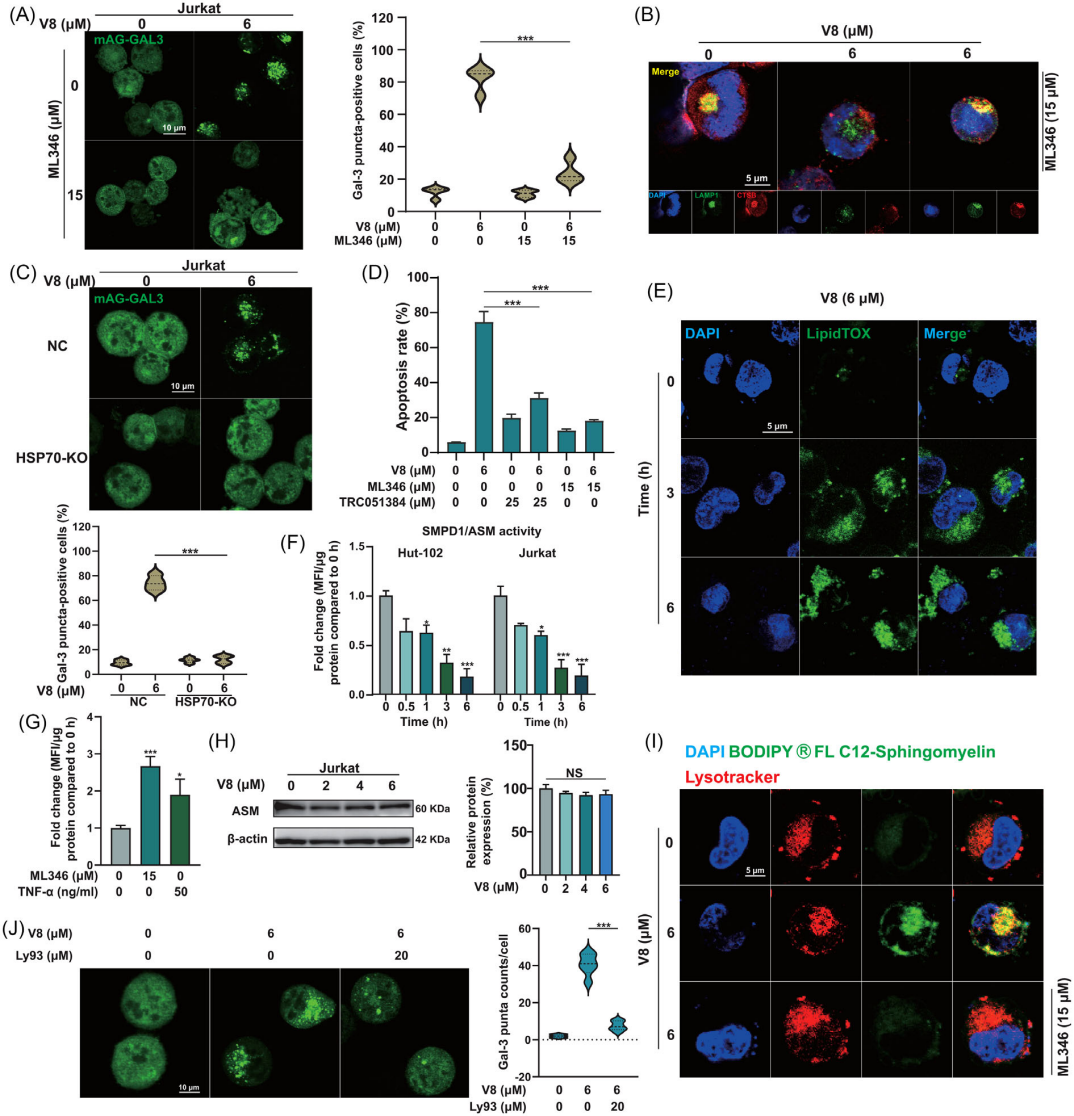
V8 disrupted lipid metabolism and disordered lysosomal membrane stability mediated by HSP70‐ASM axis. (A) Jurkat cells transfected with mAG‐GAL3 plasmid were treated with 6 μM V8 and 15 μM ML346 (pretreated for 1 h) for indicated times and the ratio of Gal‐3 puncta‐positive cells were calculated (scale bar: 10 μm) (*N* = 3). (B) Hut‐102 cells were treated with 6 μM V8 and 15 μM ML346 (pretreated for 1 h) for 6 h. Analysis of immunofluorescence performed with LAMP1 (green), CTSB (red) and DAPI (blue). An analysis of overlay levels was conducted (scale bar: 5 μm) (*N* = 3). (C) Control and HSP70‐KO Jurkat cells transfected with mAG‐GAL3 plasmid were treated with 6 μM V8 for indicated times and the ratio of Gal‐3 puncta‐positive cells were calculated (scale bar: 10 μm) (*N* = 3). (D) Apoptosis rates (%) were determined in Jurkat cells treated with 6 μM V8 and 15 μM ML346 or 10 μM TRC051384 (pretreated for 1 h) compared with control via flow cytometry labelling with Annexin V/PI (*N* = 3). (E) Hut‐102 cells were stained using the LipidTOX phospholipidosis Green staining indicates the buildup of undigested phospholipids. Nuclei were stained with DAPI (*N* = 3). (F) Assessment of ASM activity upon treatment with 6 μM V8 for 0, 0.5, 1, 3, 6 h in Hut‐102 and Jurkat cells. The bar chart represents the fold‐change of mean fluorescent intensity (MFI) per μg protein compared to control (*N* = 3). (G) Assessment of ASM activity upon treatment with 15 μM ML346 and 50 ng/ml TNF‐α for 3 h in Jurkat cells. The bar chart represents the fold‐change of mean fluorescent intensity (MFI) per μg protein compared to control (*N* = 3). (H) Jurkat cells treated with 0, 2, 4, 6 μM V8 for 6 h. Western blot was used to detect the protein expression of ASM. β‐actin was used as loading controls (*N* = 3). (I) Hut‐102 cells were co‐incubated 6 h with either dissolved in DMSO + BODIPY FL C12‐Sphingomyelin or 6 μM V8+1 μM BODIPY FL C12‐Sphingomyelin (pretreated with or without 15 μM ML346). Nuclei were stained with DAPI and Lysosomes with Lysotracker Red. Scale bar 5 μm (*N* = 3). (J) Jurkat cells transfected with mAG‐GAL3 plasmid were treated with 6 μM V8 and 20 μM Ly93 (pretreated for 1 h) for indicated times, and the ratio of Gal‐3 puncta counts/cells was calculated (scale bar: 10 μm). Three parallel experiments have yielded the mean (mean ± SEM, *N* = 3). * *p* < .05; ** *p* < .01; *** *p* < .001, compared with control group.

In order to further explain that the binding of HSP70 is the mechanism of V8 efficacy, agonists and HSP70‐KO were used to verify lysosome and survival. HSP70 agonist ML346 inhibited V8‐induced lysosomal membrane damage (Figure 5A) and LMP (Figure 5B). In HSP70‐KO cells, lysosomal membrane damage induced by V8 also decreased observably compared to NC cells (Figure 5C). Besides, HSP70 agonists ML346 and TRC051384 significant decreased V8 induced cell death (Figure 5D). It was reported that some lysosomotropic compounds could affect lipid metabolism and trigger the intracellular accumulation of phospholipids that promote lysosomal membrane damage,^45^, ^47^ based on the function of HSP70 on lysosomes we speculated V8 disturbed lipid metabolism by HSP70.^48^, ^49^ First LipidTOX phospholipidosis staining showed following V8 treatment, phospholipids were accumulated in a time‐dependent manner (Figure 5E), indicating V8 disordered lipid metabolism. As the downstream of HSP70, ASM regulates synthesis and hydrolysis of sphingomyelin (SM), which determines lysosomal fragility and LMP.^50^ SM is the second abundant phospholipids, which is the main component of cell membrane.^51^ In accordance with these reports, we confirmed a significant decrease of ASM activity in a time‐dependent manner after treated with V8 in Hut‐102 and Jurkat cells (Figure 5F). It was reported that tumor necrosis factor‐α (TNF‐α) can activate sphingomyelinase in the plasma membrane, causing sphingomyelin hydrolysis and the production of ceramide.^52^ Thus, 50 ng/ml TNF‐α was used as a positive control to increase the activity of ASM, compared with the same effect of ML346 (Figure 5G), confirming HSP70 could regulate the activity of ASM. It was worth mentioning that V8 functional inhibited ASM but did not change the protein expression of ASM (Figure 5H). Increased sphingomyelin levels in lysosomal membranes cause LMP to be produced and cathepsins to be released into the cytosol, resulting in cell death.^15^ Immunofluorescence assay showed the accumulation of SM induced by V8 was colocalization with Lysotracker Red, which was inhibited by ML346 (Figure 5I). When decreasing SM by using selective sphingomyelin synthase 2 (SMS2) inhibitor Ly93,^53^ the degree of V8‐induced lysosomal membrane damage was decreased (Figure 5J). It was indicated that V8 disrupted lipid metabolism in lysosomes and lead to LMP, which was caused by the inhibition of HSP70‐ASM axis.

### 3.6 V8 suppressed the growth of Hut‐102 xenograft tumours and induced LMP in vivo

**FIGURE 6:**
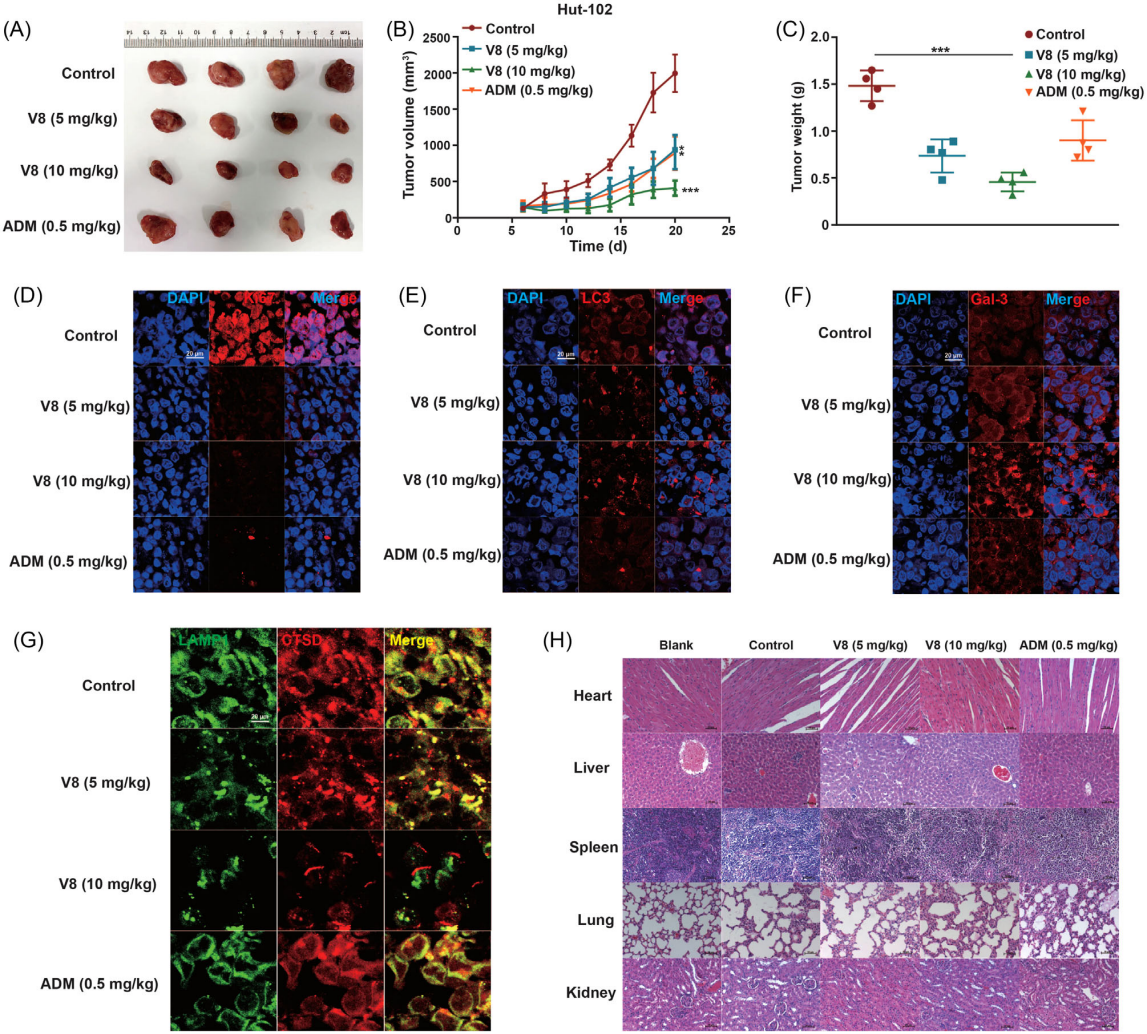
V8 suppressed the growth of Hut‐102 xenograft tumours and induced LMP in vivo. In total, Hut‐102 cells (5 × 10^6^ cells/mouse) were subcutaneously inoculated into *NOD/SCID* mice. The mice were randomized into five groups (four mice per group, blank, control, V8 [5, 10 mg/kg], ADM [0.5 mg/kg] group), and treated with 0.9% normal saline, V8 (5, 10 mg/kg), ADM (0.5 mg/kg) every 2 days for 2 weeks in control, V8 and ADM group respectively via intraperitoneal injection. Blank group is that control of observation under blank condition without any treatment. (A) The resulting tumours excised from the animals after treatment. (B) The tumour volume was measured and calculated every 2 days. (C) Three sets of tumour weights were compared. The data shown has been quantified. (D) Effect of V8 and ADM on expression of Ki67 (Red) in Hut‐102 xenografts, nuclei were stained with DAPI. Scale bar 20 μm (*N* = 3). (E) Analysis of immunofluorescence performed with LC3 (red) and DAPI (blue) in Hut‐102 xenografts. An analysis of overlay levels was conducted (scale bar: 20 μm) (*N* = 3). (F) Analysis of immunofluorescence performed with Galectin‐3 (red) and DAPI (blue) in Hut‐102 xenografts. An analysis of overlay levels was conducted (scale bar: 20 μm) (*N* = 3). (G) Analysis of immunofluorescence performed with LAMP1 (green), CTSD (red) in Hut‐102 xenografts. An analysis of overlay levels was conducted (scale bar: 20 μm) (*N* = 3). (H) H&E staining of organs in different groups (heart, liver, spleen, lung, kidney). Scale bar :50 μm. Three parallel experiments have yielded the mean (mean ± SEM, *N* = 3). ** *p* < 0.01; *** *p* < 0.001, compared with control group.

At last, to assess the anti‐tumour efficacy of V8 in vivo, we performed V8 treatment assay on the Hut‐102 xenograft model, and the *NOD/SCID* mice were administrated with 0.9% normal saline, V8 at 5 and 10 mg/kg, positive drug ADM at 0.5 mg/kg every 2 days for 2 weeks in control, V8 and ADM group respectively via intraperitoneal injection. Blank group is that control of observation under blank condition without any treatment. Compared to control, 10 mg/kg V8 resulted in a considerable decrease in tumour growth (Figure 6A–C). Moreover, decreased Ki67 positive cells in V8 treatment group showed V8 significant inhibited tumour cell growth (Figure 6D). In addition, tumour tissue immunofluorescence showed the increased LC3 and Gal‐3 puncta in V8‐treatment group (Figure 6E,F), indicating V8 induced lysosomal membrane damage in vivo. In order to confirm V8‐induced LMP in vivo, tumour tissue immunofluorescence showed LAMP1 and CTSD colocalization were decreased in 10 mg/kg group of V8 treatment (Figure 6G). For investigating whether V8 had obvious toxicity on mice, H&E staining of main organs and mice blood routine examination were used to assess the toxicity of V8, results showed V8 exerted low toxicity and had good safety (Figure 6H, Figure S5). In conclusion, V8 could effectively suppressed tumour growth and had low toxicity in vivo.

## 4 DISCUSSION

‘Lysosomotropic compounds’ was first created by de Duve et al. to define substances that accumulate in the compartments of late endosomal/lysosomal at hundreds of times greater concentrations.^54^ The accumulation in acidic lumen is also called lysosomotropism or lysosome ion trapping.^55^ The weakly basic properties of lysosomotropic compounds are owing to the chemical structure of amine group.^44^ The degree of lysosome trapping depends on the chemical properties of the drug such as pKa>7.4 and the value of clog*P* between 2 and 9.^55^, ^56^ In acidic cell compartment lumens, lysosomotropic agents accumulate, contributing to disrupt lipid metabolism balance.^43^ As a result, various sphingolipids accumulate in lysosomes and disorder the process of autophagy, affect the normal function of lysosomes. When compared to normal cells, tumour cells have more and larger lysosomes, as well as increased cathepsin activity.^45^ Here, we identified a lysosomotropic compound V8, which induced lipid accumulation, leading to lysosomal membrane damage, the inhibition of autophagic flux and LMP eventually contributes to LCD in T cell malignancies. In terms of mechanism, V8 inhibited HSP70, resulting in a decrease in ASM activity, which increased lysosomal fragility and induced the leakage of cathepsins to cytoplasm. Baf A1 inhibited the accumulation of V8 and its downstream effect because the v‐ATPase functions are to maintain acidity of acidic organelles, the pH of the lumen determine accumulation of protonated amine in the acidic organelle.

Changes in ROS or lipid composition of lysosomal membrane are the main causes of lysosomal membrane damage and LMP.^57^, ^58^ The most extensively studied mechanism of LMPs is ROS mediated LMPs.^59^, ^60^ ROS species can easily diffuse to lysosomes and interact with free intralysosomal iron, producing highly reactive hydroxyl radicals in Fenton‐type reactions and inducing LMP by causing lysosomal membranes lipid peroxidation and disrupting lysosomal membrane proteins.^42^, ^58^, ^61^, ^62^ Thus, we detected ROS level after V8 treatment, but the results showed ROS level had a slight decrease at the concentration of 4 and 6 μM of V8 (Figure 3I). However, only the increased ROS level could contribute to LMP. Thus, we excluded this factor for LMP. Moreover, we found both knockdown *CTSB* and *CTSD* could decrease V8 induced apoptosis and partially inhibited the level of LMP, but the ratio of Gal‐3 puncta‐positive positive cells could not be inhibited (Figure 3G,H,J,K). It was clear that CTSB/CTSD played its role of pro‐apoptotic in cytoplasm. When the expression of CTSB and CTSD were decreased, the level of apoptosis and LMP decreased accordingly. But with regard to lysosomal membrane damage, obviously, CTSB and CTSD are not the main cause, and it does not play a pro‐apoptotic role in acidic lumen after V8 treatment. CTSB/CTSD is a type of cathepsins with strong activity. Leakage into the cytoplasm will destroy cell homeostasis and produce the effect of cell killing. CTSB/CTSD has a potential destructive threat to the lysosome of tumour cells, which is inherently unstable.^16^ Therefore, we designed the above experiments to verify the role of CTSB/CTSD in drug effects and confirmed that lysosomal membrane damage is not caused by CTSB/CTSD, there could be another reason.

As V8 was trapped in lysosomes and induced LMP, we speculated the direct target of V8 was involved in lysosomes. Therefore, DARTS experiment was conducted to find the molecular target. Although DARTS result showed a list of possible binding proteins, we tend to select proteins that are critical for lysosomal membrane stability, due to the fact that V8 had been confirmed to accumulate in the lysosome. After scores ranking and reliability analysis compared to control group, we identified HSP70 as the potential target of V8. Subsequent CETSA and BLI experiments also confirmed the binding of HSP70 (Figure 4F,G). HSP70‐KO experiment confirmed the anticancer activity of V8 is indeed through targeting HSP70 (Figure 4J). Mutations in the *SMPD1* gene for ASM produce diminished ASM activity in cells from patients with severe lysosomal storage diseases such as Niemann–Pick disease A and B that treatment with recombinant HSP70 can effectively alleviate disease, indicating that HSP70 can protect lysosomes by stabilizing ASM.^15^, ^63^ HSP70 agonist ML346 could increase ASM activity (Figure 5G), which indicated ASM was regulated by HSP70. Tumour cell lysosomes contain low ASM levels than normal cells, their lysosomal membranes are extremely delicate and vulnerable as a result.^50^ HSP70‐ASM axis inhibition by V8 raised lysosomal SM concentration, destabilizing the already vulnerable lysosomal membrane of tumour cells, adding to LMP and cell death. With the increasing storage of lysosomotropic drugs and different types of lipid, endosomes and lysosomal compartments are blocked.^45^, ^64^ However, the accumulation of SM caused by the lack of ASM is the main inhibitor of the key steps of lysosomal sphingolipid catabolism, which regulates the catabolism of other lipids, including cholesterol, and finally blocks most lysosomal functions, resulting in cell death.^43^ Therefore, it can be concluded that the disordered catabolism of SM caused by targeting HSP70‐ASM axis was the key factor of LMP induced by V8. HSP70‐KO inhibited V8 induced lysosomal membrane damage indicated HSP70 was the initiation signal to destroy lysosomes. When added HSP70 agonist ML346, V8‐induced the accumulation of SM and LCD was both decreased (Figure 5B,D,I), which was indicated SM was correlated with the target of HSP70. And the accumulation of SM induced lysosomal membrane damage was decreased by using SMS2 inhibitor Ly93 (Figure 5J). It is worth mentioning that the verification of the role of HSP70‐ASM axis in vivo will be the focus of our future work. Overall, our data highlight the potential of V8 as a modulator of HSP70‐ASM axis mediated LMP in T cell malignancies.

## 5 CONCLUSION

We identified a lysosomotropic compound and demonstrated that V8 induced lysosomal membrane damage mediated by HSP70‐ASM axis, triggering LMP and LCD in T cell malignancies. Results from this study provide insights into the development of HSP70 inhibitor that targeting lysosomes, with V8 being a potential leading compound for further development.

## CONFLICT OF INTEREST STATEMENT

The authors declare that they have no known competing financial interests or personal relationships that could have appeared to influence the work reported in this paper.

## Supporting information

Supplementry information
