# Supplementary material for: Targeting lysosomal HSP70 induces acid sphingomyelinase‐mediated disturbance of lipid metabolism and leads to cell death in T cell malignancies
Source: Clin Transl Med. 2023 Mar 23;13(3):e1229. doi: 10.1002/ctm2.1229 (PMC10036880; doi:10.1002/ctm2.1229)

**Supplementary file**

**Supporting information**

**
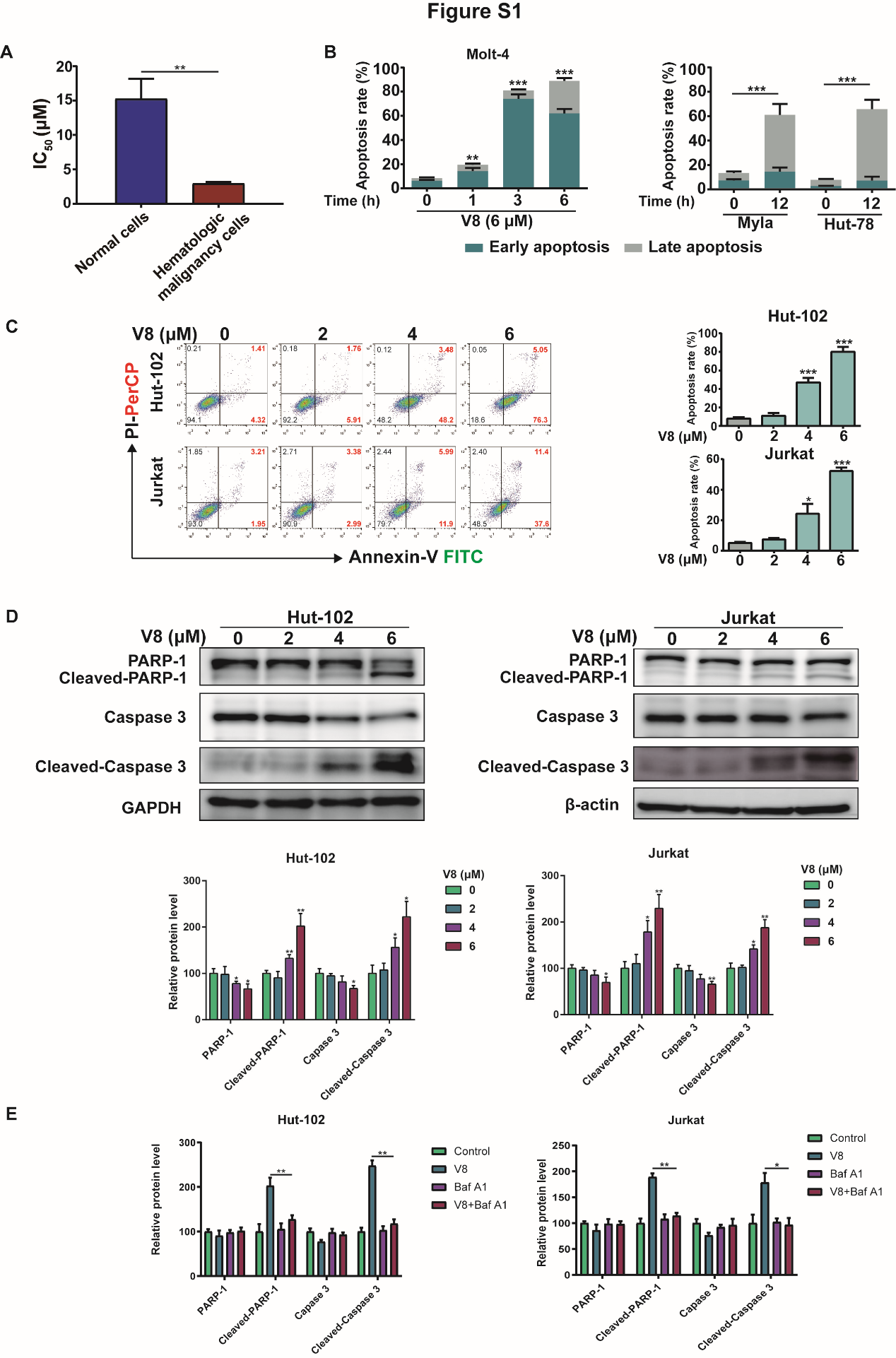
**

**Figure S1**

(A) The mean of IC_50_ in hematologic malignancy cells (Hut-102, Hut-78, Jurkat, Molt-4, Myla) and normal cells (PBMC, NCM460, HLEC, HK2, 293T). (mean ± SEM, N=5).

(B)Apoptosis rate (%) were determined in Molt-4 cells treated with 6 μM V8 for 0, 1, 3, 6 h, in Myla and Hut-78 cells treated with 6 μM V8 for 0, 12 h (N=3).

(C)Hut-102 and Jurkat cells treated with 0, 2, 4, 6 μM V8 for 6 h. Then the cell apoptosis rates were analyzed via Annexin V/PI staining by flow cytometry. Data represent the mean from three parallel experiments (mean ± SEM, N=3).

(D)The protein expression of PARP-1, Caspase-3 and its cleaved form, GAPDH, β-actin were determined by western blot. Quantitative analysis of proteins above was conducted. Data represent the mean from three parallel experiments (mean ± SEM, N=3).

(E)Hut-102 and Jurkat cells pretreated with or without Baf A1(10 nM) for 1 h, and then treated with or without V8 (6 μM) for 6 h. Quantitative analysis of the protein expression of PARP-1, Caspase-3 and its cleaved form were conducted. Data represent the mean from three parallel experiments (mean ± SEM, N=3). ******p* < 0.05, *******p* < 0.01, ********p* < 0.001, compared with control group.


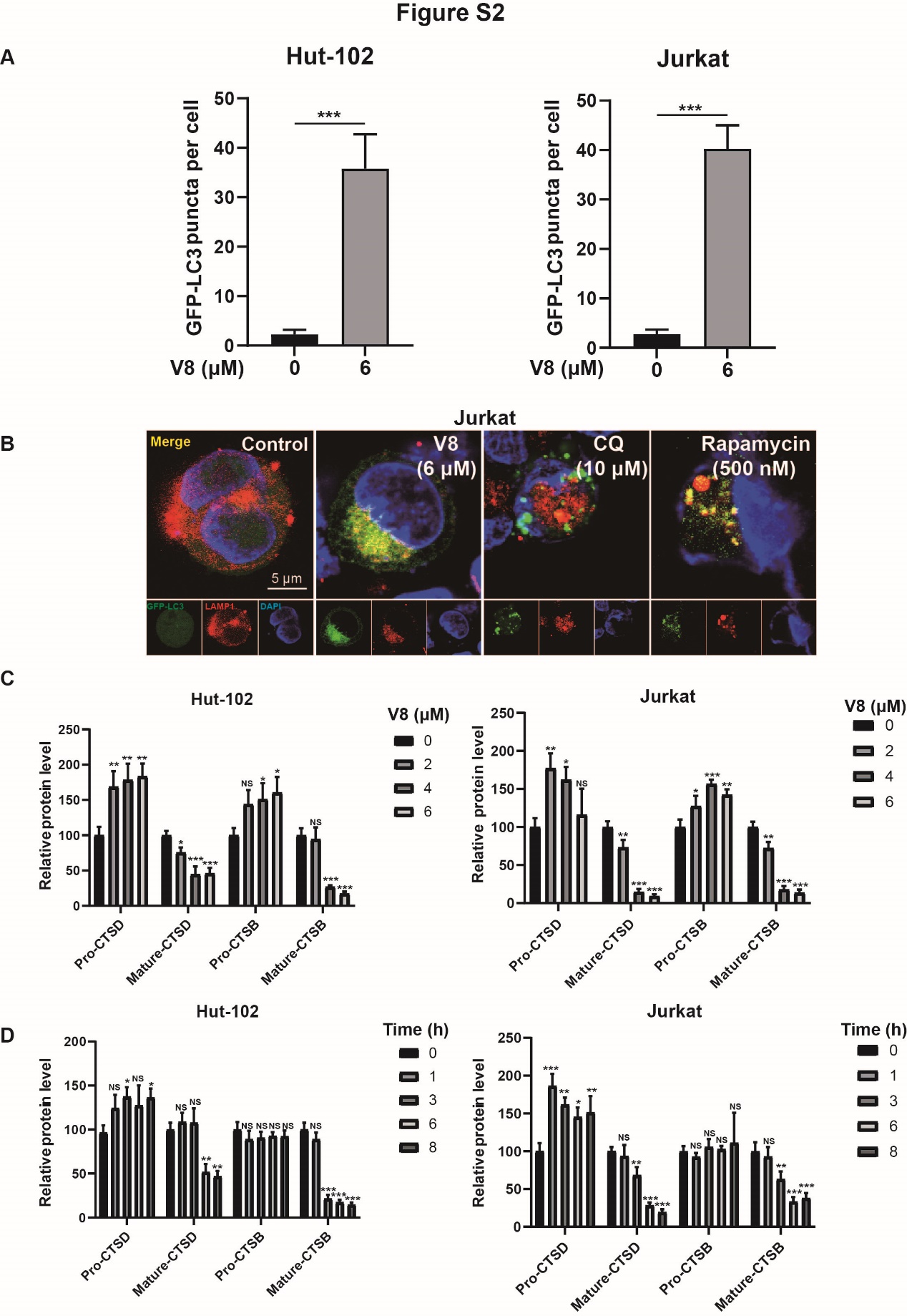


**Figure S2**

(A)The Hut-102 and Jurkat cells were transfected with a GFP-LC3 plasmid and treated with 6 μM V8 for 6 h. The GFP-LC3­ puncta per cell was quantified with image J. Data represent the mean from three parallel experiments (N=3, mean ± SEM).

(B)Jurkat cells transfected with GFP-LC3 plasmid were treated with 6 μM V8, 10 μM CQ, 500 nM rapamycin for 6 h. The immunofluorescence stained with anti-LAMP1 antibody (red; lysosomes), GFP-LC3 (green; autophagosomes) and DAPI (blue; nuclei). The overlay levels were analyzed (scale bar: 5 μm).

(C)Hut-102 and Jurkat cells treated with 0, 2, 4, 6 μM V8 for 12 h. Quantitative analysis of the protein expression of CTSB, CTSD (Pro and Mature form) were conducted. Data represent the mean from three parallel experiments (mean ± SEM, N=3).

(D)Hut-102 and Jurkat cells treated with 6 μM V8 for 0, 1, 3, 6, 8 h. Quantitative analysis of the protein expression of CTSB, CTSD (Pro and Mature form) were conducted. Data represent the mean from three parallel experiments (mean ± SEM, N=3). ******p* < 0.05, *******p* < 0.01, ********p* < 0.001, NS: no significant, compared with control group.


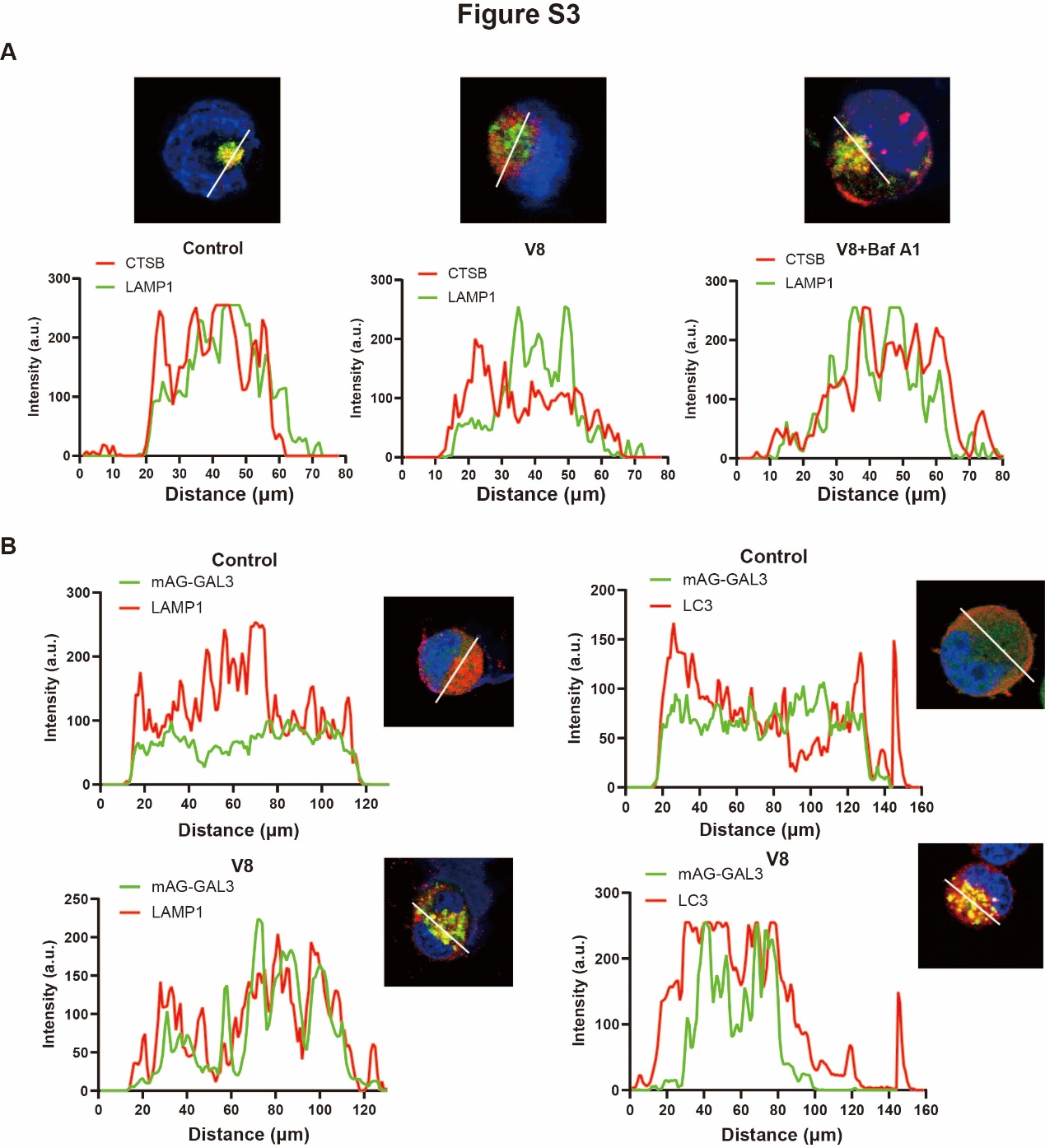


(A)The intensity profiles for both fluorescence channels of the white line positioned was measured using the Plot Profile function in the ImageJ software. The regions where the peak signal for LAMP1 (green fluorescence) and the peak signal for CTSB (red fluorescence) overlapped were identified.

(B)The intensity profiles for both fluorescence channels of the white line positioned was measured using the Plot Profile function in the ImageJ software. The regions where the peak signal for mAG-GAL3 (green fluorescence) and the peak signal for LAMP1 or LC3 (red fluorescence) overlapped were identified.


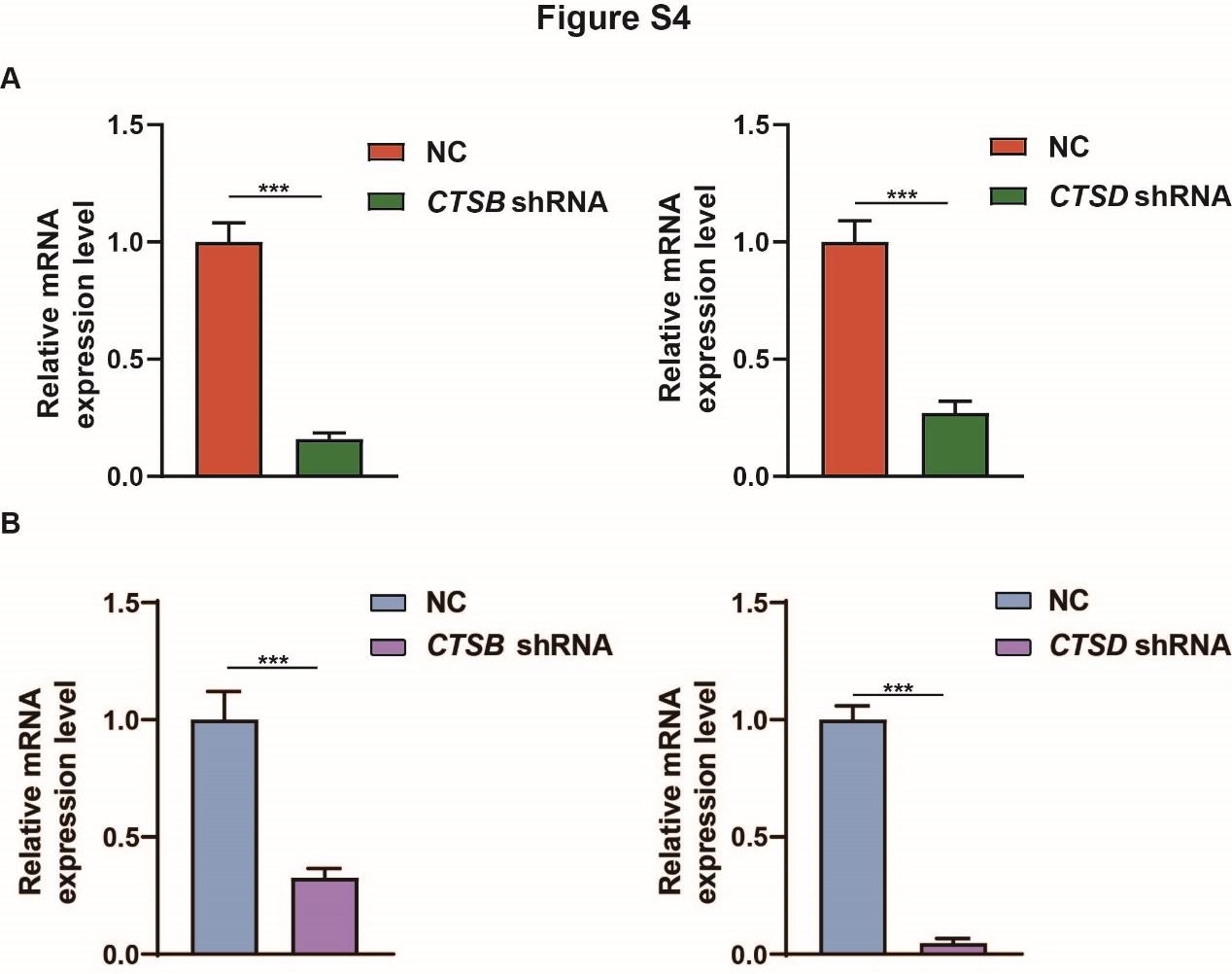


**Figure S4**

1. Relative mRNA level of *CTSB* and *CTSD* in Jurkat cells (N=3).

(B)Relative mRNA expression levels of *CTSB* and *CTSD* in DB cells. Data represent the mean from three parallel experiments (mean ± SEM, N=3). ********p* < 0.001, compared with NC group.


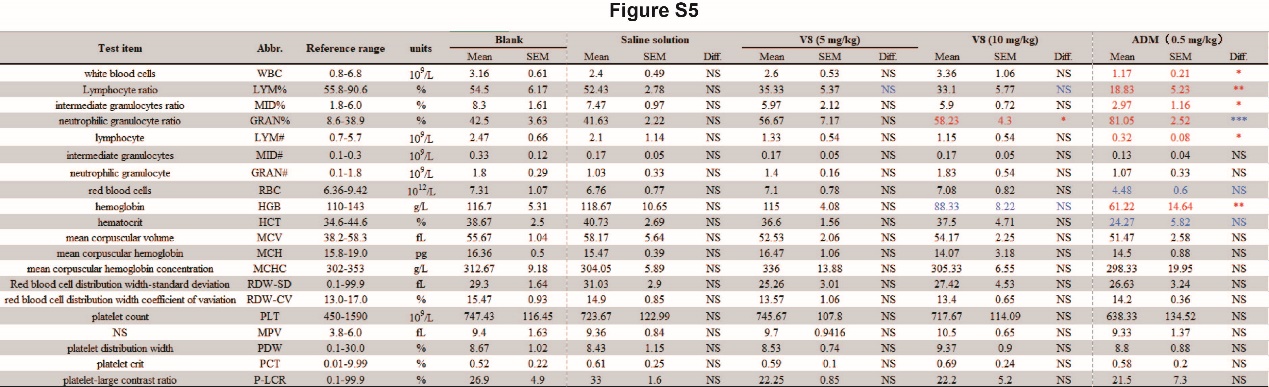


**Figure S5**

(A)Mice Routine blood test. Data are mean ± SEM, N=3, ******p* < 0.05, *******p* < 0.01, ********p* < 0.001, compared with blank group.

**Supplementary Materials and Methods**

**Determination of drug concentrations by Liquid chromatography and mass spectrophotometric (LC-MS) methods**

The concentrations of V8 in cytoplasm after lysosome separation (CLS) and lysosomal fractions were determined by LC-MS technique by following method. The analysis was performed on Agilent 1260 series LC system (Agilent technologies, USA) coupled with triple quadrupole mass spectrometer (QqQ, 6470 Triple Quadrupole) using MassHunter software. A 1 μL of sample was injected onto an Eclipse Plus C18 RRHD (1.8µm, 2.1x 50mm) (Agilent Technologies, CA, USA) and chromatographic separation occurred in methanol and water (70:30, V/V, plus 10 mM ammonium acetate) with flow rate of 0.3 mL/min at set temperature of 30℃. Operating source condition for MRM in positive ion ESI mode were optimized as follow: gas temperature 300℃, gas flow 10 L/min, nebulizer 35 psi, sheath gas temperature 350℃, sheath gas flow 11 L/min, capillary voltage 4000V , fragmentor 150V and dwell time 150 ms. The MRM modes of ions with m/z 444→160 were utilized for quantitative analysis. For the quantification of V8 in CLS and lysosome sample, a standard curve from 1-150 ng/mL of V8 was created.

**Quantitative Real-Time RT-qPCR**

The primer sequences were as follows:

*GAPDH*

Forward 5’- GGTGA AGGTCGGAGTCAACG -3’

Reverse 5’- CAAAGTTGTCATGGATGHACC -3’

*CTSD*

Forward 5’-TGCTCAAGAACTACATGGACGC-3’

Reverse 5’- CGAAGACGACTGTGAAGCACT-3’

*CTSB*

Forward 5’-GAGCTGGTCAACTATGTCAACA-3’

Reverse 5’- GCTCATGTCCACGTTGTAGAAGT-3’

*LC3*

Forward 5’- AAACGCATTTGCCATCACAGT-3’

Reverse 5’- GTGAGGACTTTGGGTGTGGTTC-3’

*SQSTM1/P62*

Forward 5’-TACGACTTGTGTAGCGTCTGC-3’

Reverse 5’- GTGTCCGTGTTTCACCTTCC-3’

**Western Blot Analysis**

Cells treated with or without V8 for certain time were collected and lysed 45 minutes on ice in RIPA buffer with protease inhibitors. Then lysates were centrifuged at 14000 rpm for 25 min at 4°C. Using BCA protein assay kit (Thermo Fisher Scientific), total protein concentrations were determined. Protein were subjected to 8%-15%. Samples were subjected to SDS-PAGE and transferred onto polyvinylidene difluoride membranes (PVDF). Membranes were blocked with 3% BSA at room temperature for one hour prior to treatment with primary antibodies overnight at 4°C. Afterwards, secondary antibodies were incubated on the membranes for 1 hour at room temperature.

**Molecular Docking**

Firstly, the ligand molecule V8 was imported and prepared by ‘LigPrep’, including ionization, desalt, generate tautomers and stereoisomers. Secondly, the protein crystal structure was imported into the software and prepared by ‘Protein Preparation Wizard’ to process the structure, including adding hydrogens, filling in missing side chains and loops using Prime, deleting waters beyond 5 Å from het groups, generating het states using Epik (pH = 7.0 ± 2.0). Thirdly, the docking sites were defined by ‘Receptor Grid Generation’. Fourthly, molecular docking was conducted in ‘Ligand Docking’ after importing prepared protein and ligand. Finally, the target HSP70-V8 complex was selected according to the docking score.

**Clustered regularly interspaced short palindromic repeats (CRISPR)/CRISPR-associated protein 9 (Cas9) genome editing**


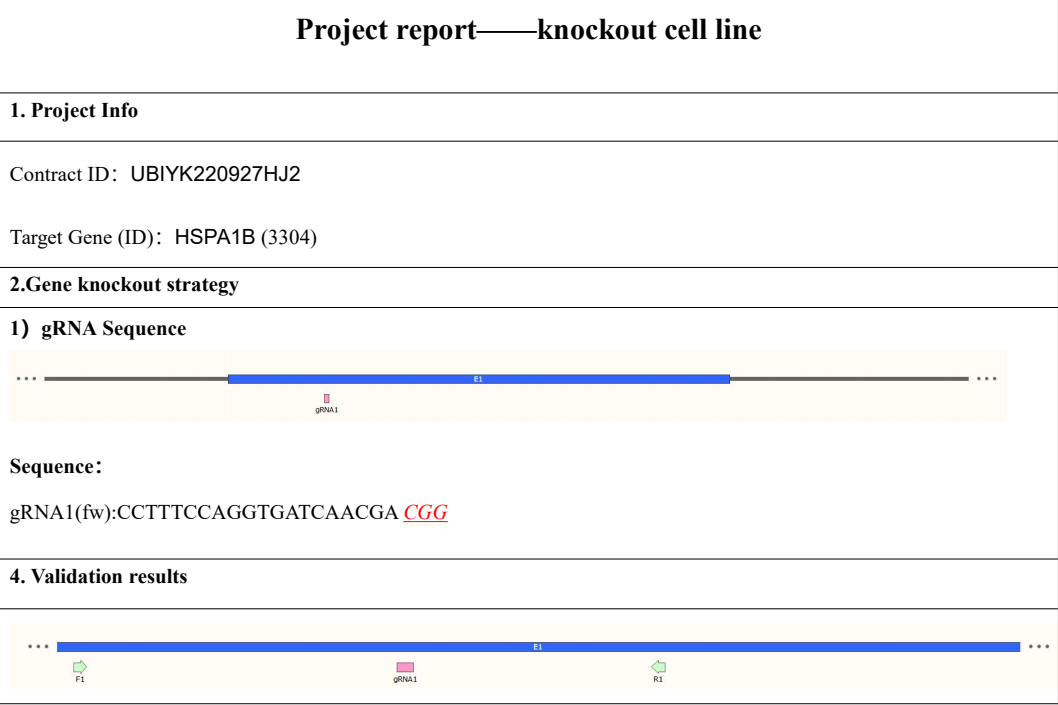


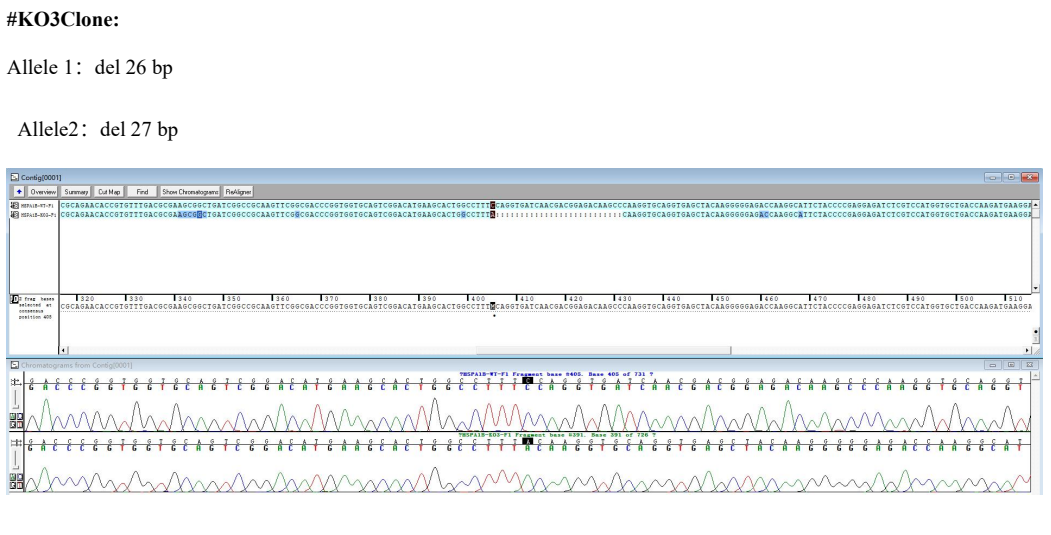


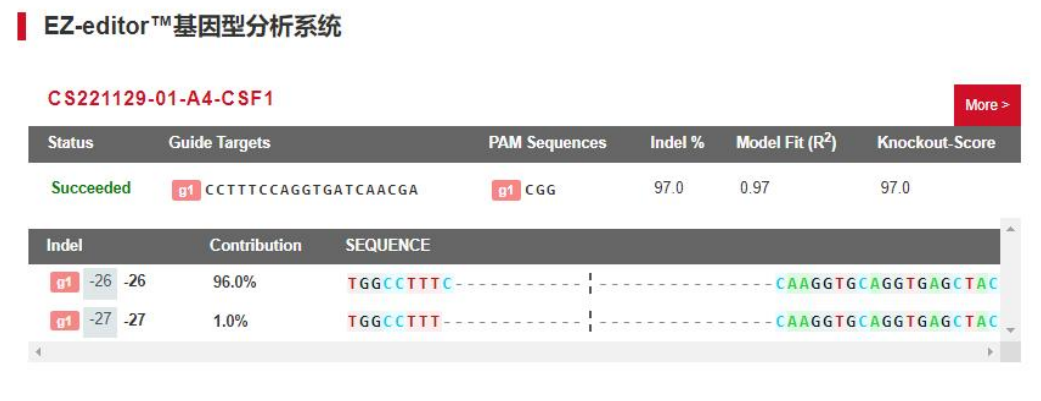

Supplement: Supplementary file 1 — Supplementry information [file CTM2-13-e1229-s001.docx]
